# Sigma1 Regulates Lipid Droplet–Mediated Redox Homeostasis Required for Prostate Cancer Proliferation

**DOI:** 10.1158/2767-9764.CRC-22-0371

**Published:** 2023-10-30

**Authors:** Halley M. Oyer, Alexandra R. Steck, Charles G. Longen, Sanjana Venkat, Konuralp Bayrak, Eleanor B. Munger, Dan Fu, Paola A. Castagnino, Christina M. Sanders, Nathalia A. Tancler, My T. Mai, Justin P. Myers, Matthew J. Schiewer, Nan Chen, Elahe A. Mostaghel, Felix J. Kim

**Affiliations:** 1 Department of Pharmacology, Physiology, and Cancer Biology, Thomas Jefferson University, Philadelphia, Pennsylvania.; 2 Sidney Kimmel Cancer Center at Jefferson, Philadelphia, Pennsylvania.; 3 Department of Chemistry, University of Washington, Seattle, Washington.; 4 Department of Urology, Thomas Jefferson University, Philadelphia, Pennsylvania.; 5 Department of Medicine, University of Washington, Seattle, Washington.; 6 Geriatric Research, Education and Clinical Center, U.S. Department of Veterans Affairs Puget Sound Health Care System, Seattle, Washington.

## Abstract

Lipid droplets (LD) are dynamic organelles that serve as hubs of cellular metabolic processes. Emerging evidence shows that LDs also play a critical role in maintaining redox homeostasis and can mitigate lipid oxidative stress. In multiple cancers, including prostate cancer, LD accumulation is associated with cancer aggressiveness, therapy resistance, and poor clinical outcome. Prostate cancer arises as an androgen receptor (AR)-driven disease. Among its myriad roles, AR mediates the biosynthesis of LDs, induces autophagy, and modulates cellular oxidative stress in a tightly regulated cycle that promotes cell proliferation. The factors regulating the interplay of these metabolic processes downstream of AR remain unclear. Here, we show that Sigma1/*SIGMAR1*, a unique ligand-operated scaffolding protein, regulates LD metabolism in prostate cancer cells. Sigma1 inhibition triggers lipophagy, an LD selective form of autophagy, to prevent accumulation of LDs which normally act to sequester toxic levels of reactive oxygen species (ROS). This disrupts the interplay between LDs, autophagy, buffering of oxidative stress and redox homeostasis, and results in the suppression of cell proliferation *in vitro* and tumor growth *in vivo*. Consistent with these experimental results, *SIGMAR1* transcripts are strongly associated with lipid metabolism and ROS pathways in prostate tumors. Altogether, these data reveal a novel, pharmacologically responsive role for Sigma1 in regulating the redox homeostasis required by oncogenic metabolic programs that drive prostate cancer proliferation.

**Significance:**

To proliferate, cancer cells must maintain productive metabolic and oxidative stress (eustress) while mitigating destructive, uncontrolled oxidative stress (distress). LDs are metabolic hubs that enable adaptive responses to promote eustress. Targeting the unique Sigma1 protein can trigger distress by disrupting the LD-mediated homeostasis required for proliferation.

## Introduction

Lipid droplets (LD) are ubiquitous, dynamic organelles that are generated *de novo* to store and traffic neutral lipids for energy and lipid precursors for membrane biosynthesis and signaling. LDs function as hubs for metabolic processes and are integral to physiologic cell metabolism (1, 2). LDs also function to sequester toxic lipid species and buffer cells from reactive oxygen species (ROS). In tumor cells, LD metabolism is co-opted to serve as an adaptive response to metabolic and oxidative stress (3, 4). LD accumulation is associated with increased cancer aggressiveness, therapy resistance, and poor clinical outcome in multiple cancers, including prostate cancer (4, 5). However, the cellular machinery and underlying mechanisms by which these LD-associated effects occur in prostate cancer remain poorly defined.

Autophagy generally describes a set of sequestration and degradation mechanisms by which cells can maintain energy levels under conditions of metabolic stress as well as a mechanism by which aggregates of misfolded proteins, lipids, and cellular components are sequestered into membrane-bound vesicles called autophagosomes and subsequently delivered to the lysosome for degradation. Several forms of autophagy have been reported, and these forms are differentiated on the basis of distinguishing mechanistic features, autophagic cargo proteins, and organelles involved (6, 7). Autophagy is an adaptive process that enables cells to cope with metabolic stress and contributes to the metabolic reprogramming and plasticity (8–11) that promotes cancer cell growth and survival (12, 13). In prostate cancer, preclinical data provide evidence that autophagy facilitates both disease progression and therapeutic resistance. By promoting metabolic capacity and plasticity and modulating oxidative stress responses (14), autophagy contributes to the adaptive resistance of tumor cells to the changing metabolic demands and stress imposed by the tumor microenvironment and cancer therapies (8–11).

The interplay between autophagy and LDs contributes to metabolic plasticity and stress adaptive responses of tumors. LDs engage in a complex bidirectional, cyclic relationship with autophagy (4). LDs have been reported to be crucial for autophagosome formation and the signaling that promotes autophagy (2, 15, 16). Reciprocally, components of the autophagy machinery are required for LD biogenesis (12, 13, 17–20). LD biogenesis is increased under conditions of high autophagic flux as a protective mechanism against lipotoxic stress (2, 21). Lipophagy describes the autophagic/lysosomal degradation of LDs (2, 7, 22). Lipophagy has been reported to promote cell growth in hormone-sensitive prostate cancer cell lines (12, 13). Other studies report that lipophagy restricts tumorigenesis (23, 24). Although it is becoming increasingly clear that aberrant metabolism and metabolic plasticity are hallmarks of cancer, we are just beginning to understand the role of LDs and their regulation by lipophagy in cancer (25).

As with LD metabolism and autophagy, redox homeostasis is crucial to tumor growth, proliferation, and survival. ROS have both productive and counterproductive effects on cells. While high levels are toxic, in limited and localized quantities ROS mediate cellular signaling that promotes survival and proliferation of cancer cells (26–30). LDs and autophagy promote redox homeostasis. LDs buffer against oxidative stress in cancer cells and support survival (4). ROS and autophagy engage in bidirectional coregulation. Moderate ROS levels can promote autophagy and prosurvival functions (31). Conversely, autophagy provides a line of defense by removing oxidatively damaged proteins, lipids, and organelles (32). The mechanisms underlying the interplay between oxidative stress and autophagy and factors involved in tuning autophagy remain largely unknown (33). The dynamic and tightly regulated interaction between metabolism and redox homeostasis drives proliferation and enables adaptive resistance during disease progression. ROS are a consequence of metabolism that drive proliferation and are also a driver of metabolic processes and proliferation (34–38). This has been reported in several cancers (33, 34, 39, 40), including prostate cancer (13, 41–43). Indeed, ROS are required for androgen-induced prostate cancer cell proliferation, and they contribute to prostate cancer progression to castration resistance (40).

Androgen receptor (AR) is the primary driver of cellular metabolism that fuels growth and proliferation of prostate cancer cells by controlling expression of enzymes involved in multiple aspects of lipid metabolism, including LD accumulation, autophagy, and production of ROS (44, 45). As prostate tumors arise, lipid metabolic enzymes are aberrantly upregulated and remain elevated throughout disease progression (44). Seminal studies showed that androgens induced accumulation of LDs in the LNCaP prostate adenocarcinoma cell line (13, 46). LDs in prostate cancer cells have been reported to be induced by both AR-dependent and -independent mechanisms (5, 13, 46).

Androgens also modulate the production of ROS. Androgens via AR have been reported to increase (47, 48) and decrease (42) ROS levels in prostate cancer cells (43, 49, 50). The ROS-inducing effects of androgens are associated with AR-induced metabolism and activation of pro-oxidative signaling pathways which stimulate prostate cancer cell growth and proliferation (47, 51). Androgen binding to AR triggers a cascade of events that produce ROS signaling and promote LD accumulation and autophagy, which in concert promote metabolic and redox homeostasis that enable prostate cancer cell proliferation (13).

The increased demand for protein production, membrane biogenesis, and *de novo* fatty acids as an energy source, render cancer cells particularly dependent on factors that maintain protein and lipid metabolism homeostasis (52, 53). Sigma1 (*SIGMAR1*; also known as sigma-1 receptor) is a unique ligand-operated chaperone or scaffolding protein that is enriched in several cancers (reviewed in ref. 54). Initially thought to be an opioid receptor, Sigma1 lacks homology with any known mammalian protein and is unrelated to any traditional receptor (reviewed in ref. 54). Emerging data suggest that Sigma1 supports the increased demand for secretory pathway protein and lipid synthesis intrinsic to cancer cells (54). In this regard, inhibition of Sigma1 can suppress growth, proliferation, and induce apoptosis in multiple cancer cell lines (reviewed in ref. 54). We have reported on a role for Sigma1 in both autophagy in cancer cells and the ability to pharmacologically induce selective autophagy with small-molecule Sigma1 modulators (55, 56). We also have demonstrated a role for Sigma1 in protein homeostasis and multiple mechanisms by which pharmacologic modulation of Sigma1 can regulate cancer cell growth and survival (54–57). However, a role for Sigma1 in cancer lipid metabolism and redox homeostasis has not been demonstrated.

Sigma1 regulates lipid metabolism and redox homeostasis in prostate cancer. We have shown previously that Sigma1 is a novel AR-interacting protein in prostate cancer cells. Sigma1 physically and functionally interacts with constitutively active AR variants (ARV) as well as full-length AR (57). Small-molecule Sigma1 modulators can be used to pharmacologically regulate AR protein levels, localization, and signaling (57). Our discoveries suggest that Sigma1 is a novel regulator of aberrant AR/ARV signaling in prostate cancer cells. However, the specific downstream aspects of AR-driven biology impacted by Sigma1 modulation have yet to be defined. Here, we report a central role for Sigma1 in the interplay between AR signaling, autophagy, LDs, and the maintenance of oncogenic ROS levels in prostate cancer cells. We propose that Sigma1 functions as a ligand-operated scaffolding protein that acts to connect the convergent signaling and metabolic pathways that drive prostate cancer cell proliferation. Sigma1 inhibition disrupts androgen-induced AR-mediated lipophagy and thus prevents the LD accumulation that maintains redox homeostasis, triggering metabolic and oxidative distress and inhibition of tumor cell growth and survival. Our work demonstrates a novel, pharmacologically responsive role for Sigma1 in prostate cancer metabolism and redox homeostasis.

## Materials and Methods

### Cell Lines and Cell Culture

LNCaP, VCaP, C4-2, 22Rv1, DU145, and PC-3 human prostate carcinoma cell lines were acquired directly from ATCC. C4-2B cells were acquired from MD Anderson's Characterized Cell Line Core. All cell lines were authenticated by short tandem repeat profiling. Cell lines were acquired within the past 6 years. Under standard culture conditions, LNCaP, C4-2, C4-2B, 22Rv1 cells were maintained in high-glucose RPMI1640 (Corning) supplemented with 10% FBS (Corning). VCaP cells were maintained in high-glucose DMEM (ATCC) supplemented with 10% FBS (Corning). DU145 and PC3 were maintained in normal glucose RPMI1640 supplemented with 10% FBS (Corning). For dihydrotestosterone (DHT) induction assays, cells were washed with Dulbecco's modified PBS solution (DPBS), followed by incubation for 3 days in phenol-red free Improved Minimum Essential Medium (Richter's Mod.) supplemented with 5% charcoal-stripped serum (CSS; Corning), then cultured for indicated times in 5% CSS medium containing 1 nmol/L DHT.

### Chemicals and Reagents

IPAG [1-(4-Iodophenyl)-3-(2-adamantyl) guanidine] was purchased from Tocris. Bafilomycin A1, 5a-DHT, and N-acetylcysteine (NAC) were purchased from Sigma-Aldrich. SA4503 [1-(3,4-dimethoxyphenethyl)-4-(3-phenylpropyl)piperazine dihydrochloride] was purchased from Axon Medchem. 17-AAG (17-N-allylamino-17-demethoxygeldanamycin) was purchased from SelleckChem. CM-H2DCFDA, HCS BODIPY 493/503, BODIPY 581/591 C-11, CM-H2DCFDA, and LipidTOX Red were purchased from Thermo Fisher Scientific.

### Plasmids and Short Hairpin RNA Constructs

Viral packaging plasmids (pRSV-REV, pVSV-G, pMDL-g/p-RRE) were obtained from AddGene. ATG5 short hairpin RNA (shRNA) constructs (TRCN0000151963, TRCN0000151474, TRCN0000330392), ATG7 shRNA constructs (TRCN0000007586, TRCN0000007588, TRCN0000364479), Sigma1 shRNA constructs (TRCN0000296908, TRCN0000291305, TRCN0000061010, TRCN0000061008), and a nontargeting control shRNA construct (SCH002) were obtained from Sigma-Aldrich. The FLAG-AR and FLAG-ARV7 plasmid constructs were gifts from Dr. Stephen Plymate (University of Washington School of Medicine, Seattle, WA) and have been described elsewhere (58). pEGFP-LC3 was a gift from Drs. Grazia Ambrosini and Gary K. Schwartz [Memorial Sloan Kettering Cancer Center (MSKCC), New York, NY] and has been described previously (56). Transient transfections were performed with jetPRIME transfection reagent (PolyPlus) according to manufacturer's procedures. LNCaP GFP-LC3 stable cell lines were generated using the pBABE-Puro-mCherry-EGFP-LC3B plasmid construct (Addgene 22418). Transfection was performed with jetOPTIMUS transfection reagent (PolyPlus) according to manufacturer's protocol. Cells were maintained in high-glucose RPMI (Corning) supplemented with 10% FBS (Corning) and 1 µg/mL puromycin (Thermo Fisher Scientific).

### Gene Set Enrichment Analysis

Normalized gene sets were downloaded from cBioPortal and the highest quartile *SIGMAR1* mRNA-expressing samples were compared with the lowest quartile *SIGMAR1* mRNA-expressing samples (59). Datasets from primary prostate tissue from The Cancer Genome Atlas (TCGA; ref. 60; Cancer Genome Atlas Research Network) and metastatic prostate tissue from SU2C/PCF Dream Team (61) were analyzed. Gene set enrichment analysis (GSEA) was performed using the Hallmark gene sets and 1,000 phenotype-based permutations (62). Normalized enrichment scores as shown, regardless of *P* value or FDR.

### Correlation Across Multiple Datasets

mRNA expression Z-scores for primary prostate cancer [443 samples from published datasets (60, 63, 64)] and metastatic prostate cancer [286 samples from three published datasets (61, 63, 64)] were downloaded from cBioPortal (59). Datasets used for the Pearson correlation coefficients were MSKCC: log_2_ whole transcript mRNA expression intensities, Fred Hutchinson Cancer Research Center (FHCRC): log_2_ mRNA expression intensities, TCGA: mRNA expression (RSEM) and SU2C: mRNA expression (RPKM). Pearson correlation coefficients and statistical significance thresholds based on sample size were calculated in R using the ggcorplot package, specifically the cor() and cor_pmat() functions.

### Single Gene Correlation Analysis

Primary prostate cancer data from MSKCC, FHCRC, and TCGA were downloaded from cBioPortal as normalized mRNA expression values (60, 63, 64). Z-scores were calculated for the genes of interest using z = (mRNA expression of sample − mean of mRNA expression)/SD of mRNA expression. Z-scores for *SIGMAR1* and genes of interest were plotted in Prism and Pearson correlations were calculated assuming Gaussian distribution of data.

### LD Assay and Confocal Imaging

Cells were seeded onto #1.5 (0.17 mm) borosilicate glass coverslips with 0.1 mg/mL 300,000+ MW poly-d-lysine substrate (Sigma-Aldrich) in standard media. After steroid starvation and drug treatments, the cells were washed with DPBS and fixed in 3% formaldehyde (Pierce, Thermo Fisher Scientific) for 20 minutes at room temperature. After washing with DPBS, neutral LDs were stained with LipidTOX diluted 1:200 in DPBS or BODIPY 493/503 1 µg/mL for 1 hour at room temperature. Staining was performed in a sealed humidified chamber. Cells were washed for 5 minutes with DPBS and nuclear counterstained with 1 µg/mL Hoechst 33342 (Invitrogen) in Hank's Balanced Salt Solution (HBSS) for 10 minutes. Cells were washed with DPBS and mounted onto glass slides using Prolong Diamond Antifade (Molecular Probes) and allowed to cure for 24 hours. Images were acquired using the Olympus FV1000 inverted confocal microscope using a 60 × 1.42 NA oil immersion objective at a scanning resolution of 0.051 µm/pixel. At least three randomly selected microscopic fields were taken for each condition.

### Autophagy Imaging

LNCaP (EGFP-LC3) stable cells were seeded onto #1.5 (0.17 mm) borosilicate glass coverslips with 0.1 mg/mL 300,000+ MW poly-d-lysine substrate (Sigma-Aldrich) in standard media. After steroid starvation and drug treatments, the cells were washed with DPBS and fixed with 3% formaldehyde (Pierce, Thermo Fisher Scientific) for 20 minutes at room temperature. After washing with DPBS, cells were nuclear counterstained with 1 µg/mL Hoechst 3342 (Invitrogen) in HBSS for 10 minutes. Cells were washed with DPBS and mounted onto glass slides using Prolong Diamond Antifade (Molecular Probes) and allowed to cure for 24 hours. Images were acquired using the Olympus FV1000 inverted confocal microscope using a 60 × 1.42 NA oil immersion objective at a scanning resolution of 0.051 µm/pixel in the FITC channel. At least three randomly selected microscopic fields were taken for each condition. Quantification of EGFP-LC3 punctae was performed as described elsewhere (55, 56).

### Quantification of LDs and Autophagosomes

The size and number of LipidTox-positive puncta per cell was quantified for at least three fields of view. Raw images were transformed into 8-bit images using ImageJ. A threshold mask of (30,255) was applied to each image, and the particles were analyzed with size threshold of greater than 30 nm. The ImageJ command “analyze particles” was used to determine the number of particles in the field. The number of particles was normalized to the number of cells in each field. Raw images from LipidTox and Autophagy microscopy were filtered to reduce background pixel noise using a confocal image filter. Cells with GFP expression were cropped, allowing for three subfields per field. Colocalization analysis was conducted using the JACop ImageJ plug-in (65). At least three randomly selected microscopic fields were taken for each condition.

### Oil Red O Staining and Quantification

LNCaP cells were seeded 6-well plates with #1.5 glass bottoms coated with 0.1 mg/mL 300,000+ MW poly-d-lysine substrate (Sigma-Aldrich) in standard media. After steroid starvation and drug treatments, the cells were washed with DPBS and fixed in 3% formaldehyde (Pierce) for 20 minutes at room temperature, followed by a PBS wash. Cells were then washed in 60% isopropyl alcohol prior to staining with Oil Red O staining solution (consisting of 0.15% Oil Red O in 60% isopropyl alcohol). Cells were stained for 20 minutes followed by washing in 60% isopropyl alcohol. Cells were then washed and incubated in PBS prior to imaging with an Eclipse Ts2 inverted microscope (Nikon) at 20X. To quantify Oil Red O content, cells were incubated in isopropyl alcohol for 5 minutes. The isopropyl alcohol solution was then collected and spun at 2300 RCF at room temperature. The supernatant was then collected, and absorbance read with GloMax Discover plate reader (Promega) at 490 nm.

### Stimulated Raman Scattering Microscopy

We used spectral focusing stimulated Raman scattering (SRS) to acquire hyperspectral SRS data in the C-H region, a technique that was demonstrated previously. The Insight DS+ laser (Newport) is used for SRS excitation. The Stokes output at 1,040 nm is modulated with an electro-optic modulator at 20 MHz and the pump is set at 800 nm. Both beams are chirped with dense flint glass rod to >1 ps pulse duration and then combined with a dichroic mirror (DMSP1000, Thorlabs). The spatial and temporally overlapped beams were sent into a home-built upright laser scanning microscope (FN1, Nikon) for hyperspectral SRS imaging. The time delay between the pump and Stokes is controlled by a fast motorized delay stage to acquire hyperspectral SRS data from 2,800 to 3,050 cm^−1^. A 40X Nikon water immersion objective (CFI APO 40XWI NA = 1.15) was used to focus the beams onto the sample with a total power of 80 mW. For signal detection, the Stokes beam was filtered out using a short-pass filter and the pump beam was detected using a large area silicon photodiode (Hamamatsu). The SRS signal was detected using a lock-in amplifier (Zurich Instrument).

### Nuclear-Cytoplasmic Fractionation

LNCaP cells were fractionated into nuclear and cytoplasmic fractions using the NE-PER extraction kit (Thermo Fisher Scientific). Briefly, cells were harvested, spun at 500 × *g* for 5 minutes at 4°C, and washed in PBS. A small part of the cell pellet was taken for the input fraction. The remaining pellet was resuspended in a hypotonic solution and vortexed occurring to the manufacturer's instruction. After incubating on ice, the cell membrane is lysed and vortexed, followed by centrifugation of the sample for 10 minutes at 14,000 rpm at 4°C. The cytoplasmic supernatant was collected, and the nuclear pellet was washed in PBS, followed by centrifugation for 10 minutes at 14,000 rpm at 4°C. After the wash supernatant was removed, the nuclear pellet was lysed and vortexed occurring to the manufacturer's instruction. The samples were incubated on ice and vortexed every 15 minutes over the course of 1 hour. The samples were then spun at 14,000 rpm for 10 minutes at 4°C. The supernatant nuclear fraction was collected All samples were stored at −80°C prior to processing with SDS-PAGE.

### Immunoblots and Antibodies

Cell lysis, protein extraction, SDS-PAGE, and immunoblotting were performed as described previously (56), with a few modifications. Briefly, lysates were separated on Novex Wedge Well Tris-Glycine mini gels (Thermo Fisher Scientific) and transferred to polyvinylidene difluoride membranes. The Luminata Western horseradish peroxidase (HRP) Substrate Chemiluminescence Kit (Millipore) was used to reveal immunoblotted proteins. The anti-Sigma1 antibody was generated in our laboratory as described elsewhere (56). The anti-ATG5 (D5F5U), anti-ATG7 (D12B11), anti-ATGL (2138S), anti-GFP (D5.1), anti-LC3B (D11 XP), anti-RCC1 (D15H6), anti-SQSTM1/p62 (5114S), anti-Sigma1 (D4J2E), anti-xBP1-s (D2C1F), and HRP-conjugated secondary antibodies were purchased from Cell Signaling Technology. Anti-β-actin (C4), anti-GAPDH (C65), and anti-PLIN5 (E-3) antibodies were purchased from Santa Cruz Biotechnology. Anti-AR-V7 [EPR15656] was purchased from Abcam.

### ROS Measurement

LNCaP and C4-2 cells were seeded into 6-well plates coated with 0.1 mg/mL 300,000+ MW poly-d-lysine substrate (Sigma-Aldrich) and incubated in CSS media for 3 days before being treated with 1 nmol/L DHT for 72 hours. Cells were washed twice with warm DPBS (Corning) and incubated with 2.5 µmol/L CM-H2DCFDA (Thermo Fisher Scientific C6827) for 30 minutes at 37°C. After incubation, cells were washed twice with warm DPBS and imaged (Nikon Eclipse Ts2) using the brightfield and FITC channels. Cells were counted using Adobe Photoshop and ROS signal was quantified using ImageJ. Total signal was quantified by dividing the ROS signal by the number of cells counted in the brightfield image.

### Glutathione/Oxidized Glutathione Ratios

Glutathione (GSH) and oxidized glutathione (GSSG) measurements were obtained using the GSH/GSSG-Glo Assay Kit (Promega) and Glutathione Assay Kit (Cayman Chemical Company). LNCaP and C4-2 cells were seeded into 96-well opaque microplates coated with 0.1 mg/mL 300,000+ MW poly-d-lysine substrate (Sigma-Aldrich) and incubated in CSS media for 3 days before being treated with 1 nmol/L DHT for 72 hours. On the day of the assay, manufacturer's protocol was followed, and luminescence was read at 1, 0.5, and 0.3 seconds with the GloMax Discover plate reader (Promega).

### C4-2 Xenograft Studies

C4-2 cells were seeded into 175 cm^2^ flasks and infected with control (SHC002, Sigma-Aldrich) and Sigma1 shRNA #4, #5 (TRCN0000061010, TRCN0000061008, Sigma-Aldrich). Cells were washed once with DPBS (Corning), detached with trypsin (0.05% Trypsin/EDTA, Corning), and pelleted at 500 × *g* for 5 minutes. After resuspending cells in DPBS, an equal volume of Matrigel Matrix (Corning) was added to the cell slurry. BALB/c Nude mice (Charles River) were anesthetized and injected with the cell slurry on their right and left flanks. Viability at injection was determined using 0.4% Trypan Blue Stain (Invitrogen). Mice were monitored daily for 1 week and tumor volume measurements were taken each week after for 11 weeks. Organs were harvested on the 12th week of the study, and subsequently Oil-Red-O and hematoxylin and eosin staining were performed.

### Statistical Analysis

For all experiments, at least three biological replicates were performed. Immunoblots were normalized to the loading control then appropriate control within the experiment. Comparisons between two groups were analyzed on GraphPad Prism using a two-tailed, unpaired *t* test, and multiple groups using Tukey multiple comparisons test.

### Data Availability Statement

The data analyzed in this study were obtained from cBioPortal for Cancer Genomics at SU2C/PCF Dream Team, MSKCC, FHCRC, and TCGA. Hallmark Gene Sets were obtained from GSEA Molecular Signatures Database (GSEA MSigDB) at Hallmark Gene Set.

## Results

### Sigma1 is Required for LD Accumulation in Prostate Cancer Cells

**FIGURE 1:**
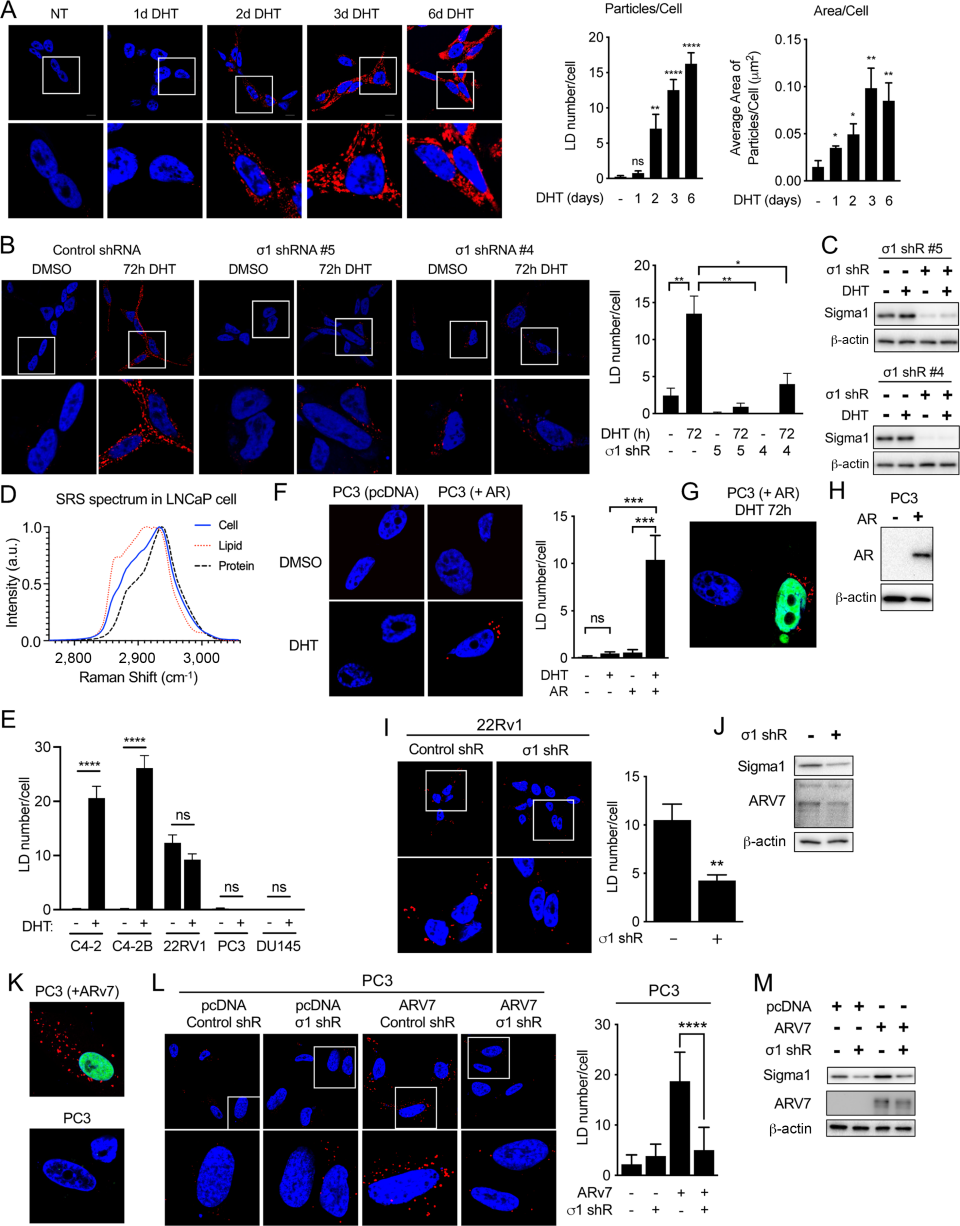
Sigma1 is required for DHT-induced AR-mediated LD accumulation in prostate cancer cells. **A,** Confocal micrograph showing LD accumulation in LNCaP cells cultured in CSS containing medium for 3 days and then treated for 1, 2, 3, and 6 days of DHT (1 nmol/L). HCS LipidTOX stained LDs (red). DAPI stained nuclei (blue). Quantification of LD number per cell and average area of LD particles/cell. Data represent mean values from at least three independent determinations, and error bars represent SEM. LD particle numbers and lipid area were quantified using ImageJ. Statistical analysis was performed using ANOVA and Bonferroni after test. *, *P* < 0.05; **, *P* < 0.01; ***, *P* < 0.001; ****, *P* < 0.0001**. B,** Confocal micrograph showing LD accumulation in LNCaP transduced with nonspecific control and Sigma1 shRNA. Two distinct Sigma1 shRNA clones were tested and produced comparable results. Cells were cultured in CSS containing medium for 3 days and then treated for 3 days with DHT (1 nmol/L). LD number per cell was determined as in A, above. **C,** Immunoblots of whole-cell protein extracts from LNCaP cells infected with Sigma1 shRNA #4 and #5 and treated, serum starved for 3 days, and treated with 3 days of DHT. **D,** SRS confirming lipid content of LDs in LNCaP cells following 3 days of 1 nmol/L DHT treatment in CSS medium, conditions described above. **E,** LD numbers per cell in panel of AR-driven (C4-2, C4-2B), ARV-driven (22Rv1), and AR-negative, independent (PC3, DU145) prostate cancer cell lines. Data represent mean values from at least three independent determinations, and error bars represent SEM. *, *P* < 0.05; **, *P* < 0.01; ***, *P* < 0.0001; ns = no significance. **F,** Confocal micrograph of LDs in PC3 cells (endogenous AR-negative prostate cancer cell line), transfected with empty vector (pcDNA) or recombinant AR plasmid, then treated with DHT (1 nmol/L, 3 days). Quantification of the average number of LDs per cell. Right, Quantification of the mean number of particles per cell ± SE. *, *P* < 0.05; **, *P* < 0.01. **G,** Confocal micrograph showing that LDs accumulate only in AR-transduced PC3 cells. AR (green), LDs (red), DAPI stained nucleus (blue). **H,** Immunoblot further confirming transduction and expression of recombinant AR in PC3 cells. **I,** ARV7-induced LDs require Sigma1. LDs (red) in 22Rv1 cells transduced with nonspecific control shRNA or Sigma1 shRNA #5. Magnified inset (white boxes) shown below. LD stain (red), DAPI stain (blue). **J,** Immunoblot confirmation of Sigma1 shRNA KD in 22Rv1 cells. **K,** Control confirming that only ARV7-positive cells are also LD-positive. ARV7 immunostain (green), LD stain (red), DAPI stain (blue). **L,** LDs (red) in PC3 cells transduced with nonspecific control shRNA or Sigma1 shRNA #5 and subsequently transfected with ARV7. Magnified inset (white boxes) shown below. DAPI stain of nuclei (blue). Average number of LDs per cell calculated and analyzed as above. **M,** Immunoblot confirmation of Sigma1 shRNA KD and transfected ARV7 expression in PC3 cells.

The androgen-sensitive, endogenous AR-expressing LNCaP prostate cancer cell line is the model originally and most widely used to describe prostate cancer cell lipid metabolism (13, 46, 66). Several published reports have shown that androgens, including DHT can induce LD accumulation in LNCaP cells (13, 46). We confirmed this effect by incubating hormone-sensitive LNCaP cells in androgen-depleted CSS medium and treating with DHT (1 nmol/L, up to 6 days). We performed confocal microscopy to visualize LDs using the fluorescent neutral lipid stain HCS LipidTOX. As described elsewhere, this neutral lipid stain has a narrower emission spectrum than the commonly used BODIPY 493/503 stain and thus is less susceptible to bleed through into other fluorescent channels and less susceptible to false-positive staining (13). This was important for our LD colocalization studies, described below. Consistent with previously published data, over a 6-day time course, we observed salient and significant accumulation of DHT-induced LDs, with total LD area per cell plateauing between 3 and 6 days (Fig. 1A).

We have published that Sigma1 physically and functionally interacts with AR (57). We also demonstrated that inhibition of Sigma1 suppresses aberrant AR signaling in prostate cancer cells (57). Therefore, we asked whether androgen-induced, AR-mediated LDs are Sigma1 dependent. We found that shRNA-mediated knockdown (KD) of Sigma1 in LNCaP cells abrogated DHT-induced LD accumulation (Fig. 1B). We tested two distinct Sigma1 shRNA clones and observed similar results with both. Under these conditions, approximately 80% KD of Sigma1 was achieved with both Sigma1 shRNA clones, measured by immunoblot (Fig. 1C).

To confirm that the labeled vesicular structures that decreased upon Sigma1 inhibition were indeed LDs, we performed SRS microscopy to identify and quantify their content. SRS is an emerging optical imaging technique that utilizes the intrinsic vibrational signatures of molecules to image their distributions and quantify their concentrations at subcellular resolution (67, 68). We have reported the use of SRS to quantify neutral lipid content in living cells and organisms (68, 69). Using this approach, we determined that under these experimental conditions, DHT-induced LDs contain primarily triacylglycerols with some cholesterol esters in proliferating LNCaP cells (Fig. 1D).

Swinnen and colleagues published that androgen induces neutral lipid accumulation into LDs in LNCaP cells. This effect was blocked by the AR antagonist bicalutamide in AR-expressing cells, and it was not observed in the AR-negative PC3 and DU145 cell lines, suggesting that LD induction in this context is AR mediated (46). We also found that DHT did not induce LD accumulation in PC3 and DU145 cells (Fig. 1E). Furthermore, to confirm that androgen-induced LDs are indeed AR dependent, we treated PC3 cells (which do not express endogenous AR) transfected with an empty vector or with an AR expression vector with DHT, and we detected LDs only in PC3 cells that expressed the transfected AR (Fig. 1F–H). Consistent with these data, we also found that LDs did not accumulate in DU145 cells, regardless of DHT treatment (Fig. 1E). As with LNCaP cells, DHT induced LD accumulation in C4-2 and C4-2B cells (Fig. 1E). Altogether, these data demonstrate that Sigma1 and AR are both required for DHT-induced LDs.

Interestingly, LD accumulation was observed in the constitutively active ARV-driven 22Rv1 cell line, with similar numbers of LDs with or without DHT, suggesting that LD accumulation in 22Rv1 cells was induced by ARV (Fig. 1E). Importantly, LDs were present in 22RV1 cells grown in hormone-depleted CSS medium, conditions in which full-length AR would be minimally active or inactive. KD of Sigma1 eliminated LDs in 22Rv1 cells, suggesting a role for Sigma1 in ARV-driven LD accumulation in these cells (Fig. 1I and J). 22Rv1 cells express the clinically relevant, constitutively active ARV7. The specificity of androgen-independent, ARV7-mediated LD accumulation was confirmed in PC3 cells grown in CSS medium and transfected with a recombinant ARV7 expression vector. The number of LDs per cell significantly increased only in ARV7-transfected PC3 cells (Fig. 1K). As with 22Rv1 cells, Sigma1 KD prevented the formation of LDs in ARV7-transfected PC3 cells (Fig. 1L and M). These data demonstrate that both full length and constitutively active ARV7 can induce LDs in a hormone-independent, Sigma1-dependent manner. These results are consistent with our previous discovery that Sigma1 interacts with and can alter the signaling of ARVs as well as full-length AR (57).

### Sigma1 KD Prevents Accumulation of LDs by Triggering Lipophagy

**FIGURE 2:**
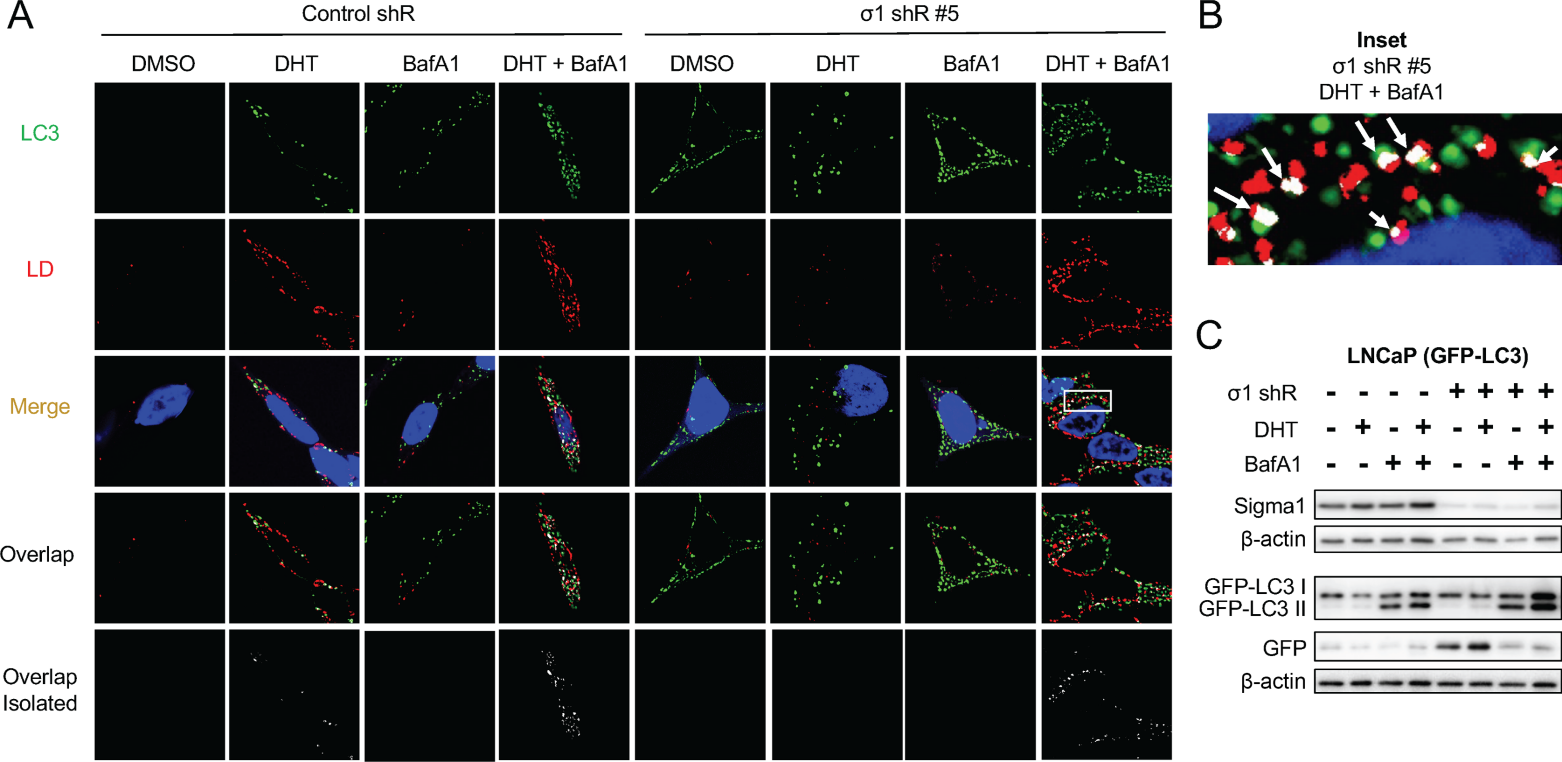
Sigma1 KD triggers lipophagy. **A,** Confocal micrographs showing colocalization of GFP-LC3 (LC3, green) and HCS LipidTox labeled LDs (red) in Sigma1 shRNA-transduced LNCaP (GFP-LC3) cells that were serum-starved for 3 days and treated with 1 nmol/L DHT for 3 days alone or combined with 10 nmol/L bafilomycin A1 (BafA1) for the final 8 hours prior to fixing the cells. **B,** Inset from column 8, Merge from A showing magnified view of autophagosome (LC3, green) colocalization with LD (red). Overlapping, colocalization events indicated by white arrows. **C,** Immunoblots of whole-cell protein extracts from parallel cell culture performed in parallel and using same experimental treatment conditions. GFP, GFP-LC3 I, and GFP-LC3 II were detected using an anti-GFP antibody. GFP-LC3 II band represents autophagosomes, similarly to canonical LC3B II immunoblot banding patterns. Independent GFP band indicates active autolysosomal degradation, autophagic flux.

Sigma1 is a multifunctional protein, and it has multiple activities beyond its interaction with AR signaling (54). Indeed, we previously published that pharmacologic Sigma1 modulation could trigger autophagy (55, 56). Here we observed that Sigma1 KD triggered autophagy and autophagosomal degradation of DHT induced LDs in a manner consistent with lipophagy (Fig. 2). We stably transfected LNCaP cells with a GFP-LC3 construct [described elsewhere (55)] and generated LNCaP (GFP-LC3) cells to evaluate the role of lipophagy as a potential mechanism by which Sigma1 KD prevents DHT-induced LD accumulation. We performed confocal microscopy to detect LDs, autophagosomes (GFP-LC3 labeled autophagosomes) and monitor their colocalization. In nonspecific control shRNA-transduced LNCaP (GFP-LC3) cells, DHT (1 nmol/L, 3 days in CSS medium) induced salient LD accumulation with modest induction of autophagosome formation (Fig. 2A). We detected modest colocalization of the autophagosome (LC3) and LD signals, suggesting low levels of DHT-induced lipophagy (see bottom row of second column from left in Fig. 2A). To confirm that colocalized LDs and GFP-LC3 labeled autophagosomes were undergoing autophagic flux (lysosomal degradation of LD containing autophagosomes) we added the widely used vacuolar H+-ATPase inhibitor Bafilomycin A1 (BafA1), which blocks autophagosome-lysosome fusion and thus blocks autolysosomal degradation or autophagic flux (7, 70), during the final 4 hours of the 3-day DHT treatment to block autophagic flux at the autolysosome stage. We observed a clear increase in colocalization of LDs and autophagosomes (GFP-LC3) in the DHT + BafA1 condition, demonstrating complete lipophagy, including flux or the degradation of LDs by autophagy (fourth column from left in Fig. 2A).

Next, we asked whether shRNA KD of Sigma1 prevented LD accumulation by triggering lipophagy. We discovered that Sigma1 KD triggered a salient induction of autophagosomes, however, with no increase in LDs [compare DMSO (drug vehicle controls) column 1 and 5 of Fig. 2A]. Consistent with the results in Fig. 1, LDs did not accumulate in DHT-treated LNCaP (GFP-LC3) cells in which Sigma1 was knocked down (column 6 of Fig. 2A). When BafA1 was added to LNCaP (GFP-LC3 Sigma1 shRNA KD) cells during the final 4 hours of the 3-day DHT treatment of cells, we observed a salient increase in LD accumulation and their colocalization with autophagosomes (column 8 of Fig. 2A and B). We quantified LD-autophagosome colocalization events in the DHT, DHT + bafilomycin A1, and DHT + bafilomycin A1 in Sigma1 shRNA KD cells using Mander coefficient overlap correlation analysis (71), and we confirmed significant differences in overlap coefficient in these treatment conditions where increased LD and autophagosome colocalization was observed (Supplementary Fig. S1). We confirmed autophagic flux using an assay in which the cleavage of GFP-LC3 and release of GFP is an indicator of autolysosomal degradation (Fig. 2C). We have described this assay in the context of Sigma1 modulation in detail elsewhere (55, 56). Altogether, these results suggest increased autophagic flux in Sigma1 KD cells and lipophagy as a key mechanism by which DHT-induced LDs are eliminated in prostate cancer cells.

In addition, and as anticipated, preventing the formation of autophagosomes by shRNA KD of essential autophagy genes ATG5 and ATG7 also prevented formation of LDs (Supplementary Fig. S2). This was consistent with other publications showing that the autophagy machinery is required for LD formation. LDs have been reported to be required for autophagosome formation (16, 72). Reciprocally, it has been reported that components of the autophagy machinery (MAP1-LC3, ATG5, ATG7) are required for LD biogenesis; MAPLC31 in prostate cancer cells (12, 13) and hepatocytes (17), *ATG5* in mouse embryonic fibroblasts (18), ATG5 and 7 in adipocytes (20). Here, we showed that ATG5 and ATG7 are required for androgen-induced LDs in prostate cancer cells as KD of ATG5 and ATG7 prevented the formation LDs (Supplementary Fig. S2). Our results confirm and extend evidence supporting the notion that LD and autophagy machineries are interdependent.

### Sigma1 KD Suppresses Prostate Cancer Cell Proliferation *In Vitro* and Tumor Growth *In Vivo* Despite Lipophagy

**FIGURE 3:**
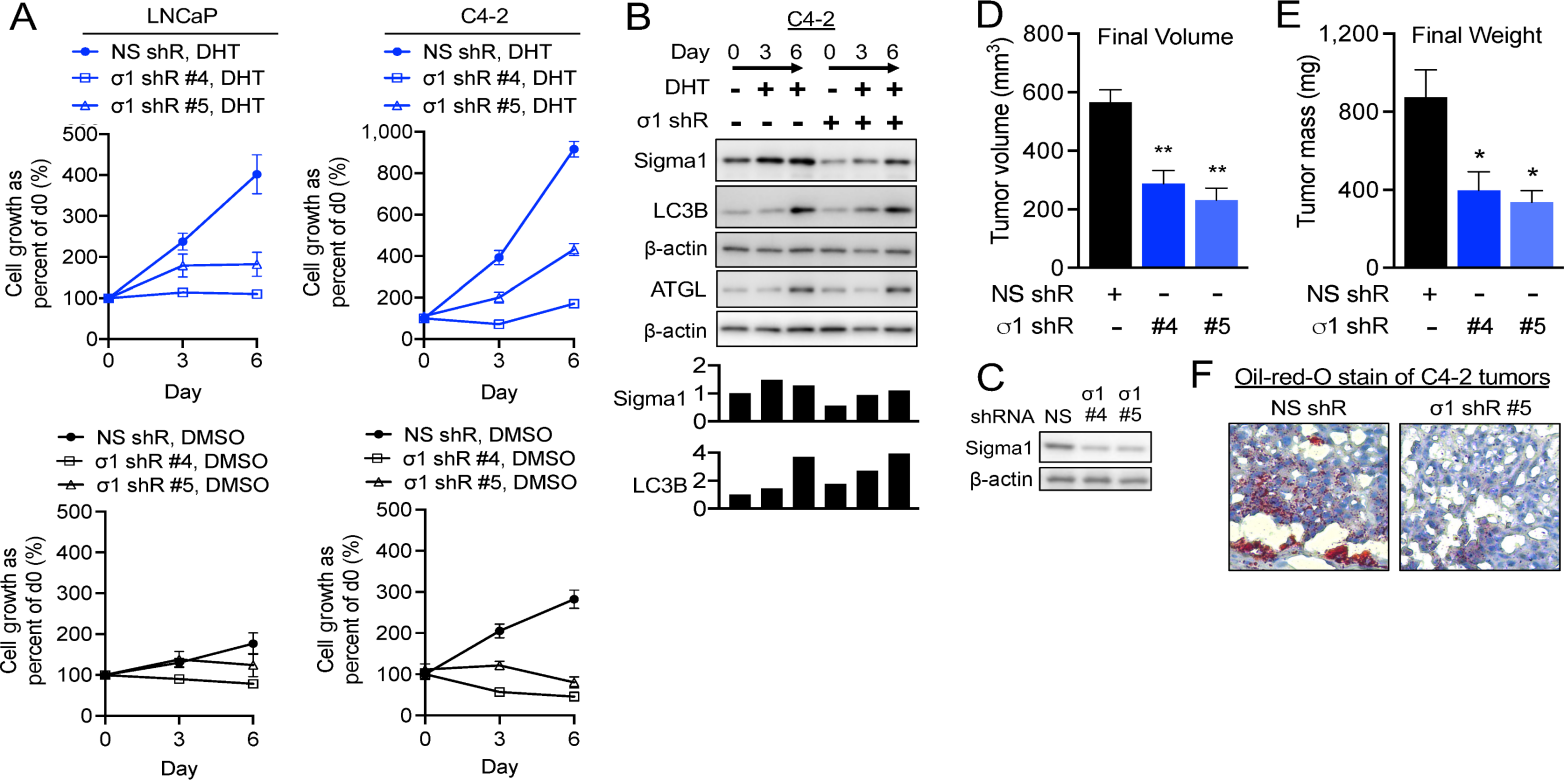
Sigma1 KD suppresses DHT induced prostate cancer cell proliferation and tumor growth despite (or due to) increased autophagy. **A,***In vitro* cell proliferation assay of Sigma1 shRNA-transduced LNCaP and C4-2 cells precultured in CSS medium for 3 days and then treated for 3 and 6 days with 1 nmol/L DHT. Live cells were counted by Trypan blue exclusion assay at the start of the time course (day 0), and 3 and 6 days of DHT treatment. Data are represented as fold induction over cells treated with control shRNA at day 0. Datapoints represent mean fold increase in cell number from at least three independent determinations, and error bars represent SEM. Two distinct Sigma1 shRNA clones, #4 and #5, were tested and produced similar results. **B,** Immunoblots of whole-cell protein extracts from parallel C4-2 cell culture performed in parallel and using same experimental treatment conditions as in A. Data shown for Sigma1 shRNA clone #5 KD C4-2 cell cultures. **C,** Immunoblot of Sigma1 shRNA clone #4 and #5 transduced C4-2 cells immediately prior to subcutaneous flank implantation into SCID mice. **D,** C4-2 cells infected with Sigma1 shRNA (#4, #5) and control shRNA (#1) were injected into the right and left flanks of SCID mice. Tumor volume was measured by caliper 12 weeks after implantation, prior to sacrificing the mice. Data are represented as mean volume of six tumors for each condition, and error bars represent SEM. **E,** Tumor weight was measured at 12 weeks postinjection at the time of harvest. Data are represented as mean volume of six tumors for each condition, and error bars represent SEM. *, *P* < 0.05; **, *P* < 0.01. **F,** Oil Red O staining of control (clone #1) and Sigma1 shRNA (clone #4 and #5) xenografted C4-2 tumors.

Autophagy, and lipophagy more specifically, has been reported to promote prostate cancer proliferation and survival (4, 13). We asked how Sigma1 KD-associated lipophagy would impact prostate cancer cell proliferation *in vitro*. LNCaP and C4-2 cells were cultured in medium containing CSS supplemented with 1 nmol/L DHT for 6 days. Two distinct Sigma1 shRNA clones were compared with nonspecific control shRNA in both cell lines. Sigma1 KD in LNCaP, C4-2, and VCaP cells significantly suppressed proliferation *in vitro*, despite corresponding increase in lipophagy (Fig. 3A and B for LNCaP and C4-2, and VCaP data shown in Supplementary Fig. S3).

Furthermore, Sigma1 shRNA KD in xenografted C4-2 tumors (Fig. 3D) resulted in significantly decreased tumor volume and corresponding decrease in tumor weight (Fig. 3E). An approximately 50% Sigma1 KD resulted in proportional decrease in both tumor volume and weight (Fig. 3C–E). Oil red O staining of tumors at the end of the study revealed decreased levels of neutral lipids (Fig. 3F), consistent with our *in vitro* LD data. Sigma1 KD suppressed prostate cancer cell proliferation *in vitro* and tumor growth *in vivo*. This occurred despite increased lipophagy in Sigma1 KD cells.

### LDs Buffer Against Androgen-induced ROS and Sigma1 KD Prevents Antioxidant Response

**FIGURE 4:**
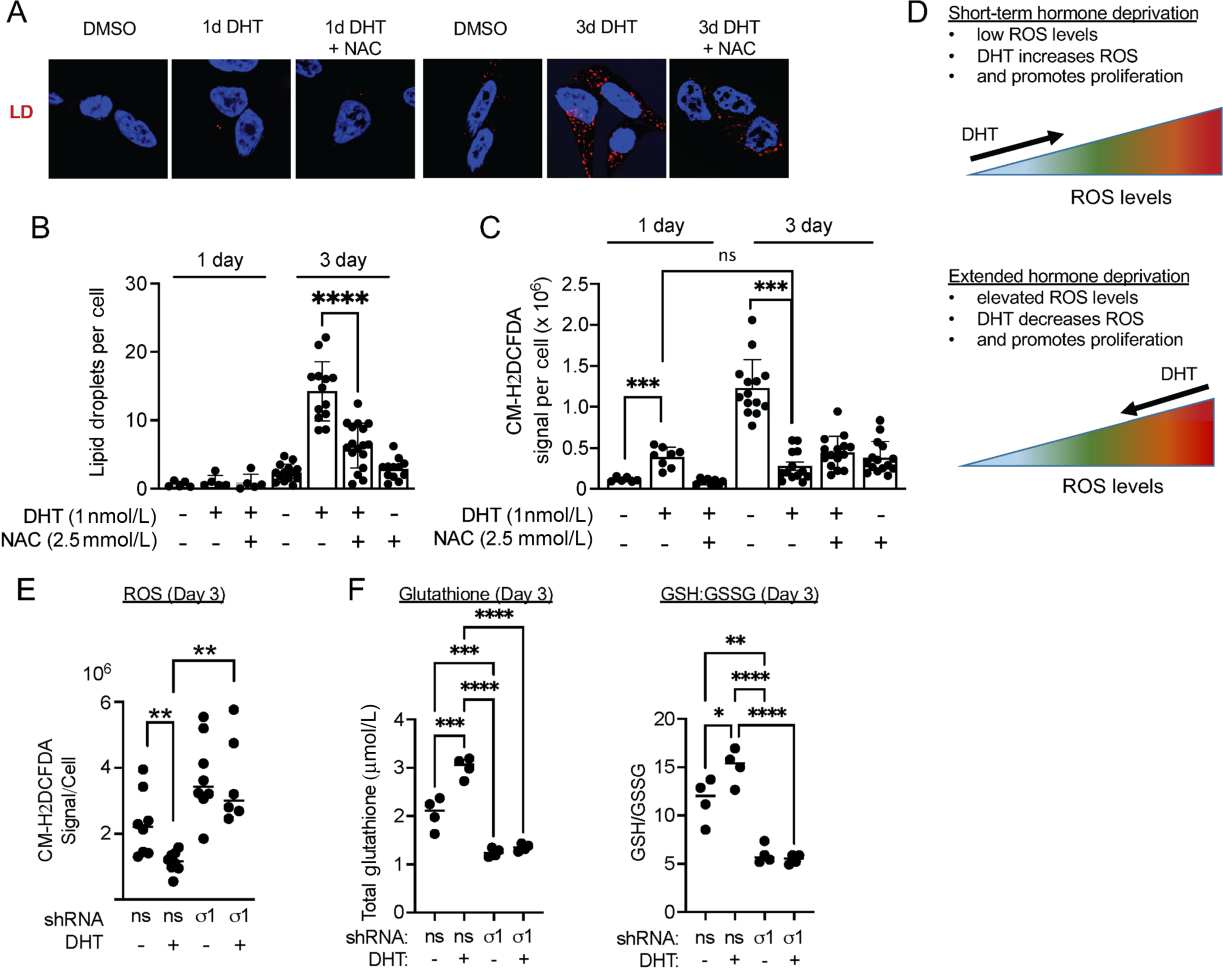
LDs as buffers of DHT induced ROS and DHT promotes ROS homeostasis. **A,** Confocal micrograph showing HCS LipidTox stained LDs in LNCaP cells cultured in CSS containing medium for 3 days and treated with DMSO (vehicle) and 1 nmol/L DHT alone or combined with 2.5 mmol/L NAC for 3 days. **B,** LD quantification of LNCaP cells from A. Data represent LDs per cell and error bars represent SEM. *, *P* < 0.05; **, *P* < 0.01; ***, *P* < 0.001; ****, *P* < 0.0001. **C,** Quantification of ROS, detected with CM-H_2_DCFDA in LNCaP treated as described above in A. Data are presented as mean ± SEM from at least three independent determinations. *, *P* < 0.05; **, *P* < 0.01; ***, *P* < 0.001; ****, *P* < 0.0001. **D,** Illustration of concept that DHT initially induces ROS to trigger proliferation (1 day of DHT), and subsequently decreases intracellular ROS levels. LD accumulation is observed as DHT decreases ROS levels. **E,** Quantification of ROS in control (#1) or Sigma1 shRNA (#5) transduced LNCaP cells treated as described above in A. Each datapoint represents mean CM-H_2_DCFDA signal per cell from three fields in three independent wells. **F,** Redox balance. Total GSH levels and ratio of GSH-to-GSSG measured in nonspecific control shRNA (ns) and in Sigma1 shRNA (#5) transduced LNCaP cells treated as described above in A.

Previous reports have suggested that androgens induce moderate (limited and localized) quantities of ROS to promote prostate cancer cell proliferation (26–30, 40), while LDs have been shown to function as buffers of ROS. (4) Consistent with these concepts, we observed an inverse correlation of ROS levels and LD numbers in DHT-treated LNCaP cells. In LNCaP cells, 3 days of culture in CSS medium resulted in increased ROS levels and low LD numbers, and by 3 days of DHT treatment, we observed a significant decrease in ROS levels (CM-H2DCFDA signal per cell) with a corresponding increase in LDs (Fig. 4A–D). Cotreatment with NAC, acetylated precursor of reduced GSH and scavenger of oxygen-free radicals, significantly decreased the number of 3-day DHT treatment--induced LDs in LNCaP (Fig. 4A and B), C4-2 (Supplementary Fig. S4A and S4B), and VCaP cells (Supplementary Fig. S4C and S4D). Altogether, these data suggest that DHT induces LDs, in part, to buffer against oxidative stress and maintain redox homeostasis.

Next, we asked whether Sigma1 was required for DHT-mediated antioxidant response. We found that shRNA KD of Sigma1 in LNCaP cells resulted in elevated ROS, and DHT treatment failed to decrease ROS levels in Sigma1 KD cells (Fig. 4E). DHT treatment increased total GSH concentrations and reduced GSH-to-GSSG ratios (GSH/GSSG) in LNCaP cells, consistent with a redox homeostasis promoting, antioxidant response induced by AR (Fig. 4F). In contrast, Sigma1 KD reduced DHT-induced total GSH and GSH/GSSG ratios, suggesting a central role for Sigma1 in redox homeostasis mediated by the androgen-AR axis (Fig. 4F).

### Small-molecule Sigma1 Inhibitor Induces Lipophagy to Prevent LD Accumulation

**FIGURE 5:**
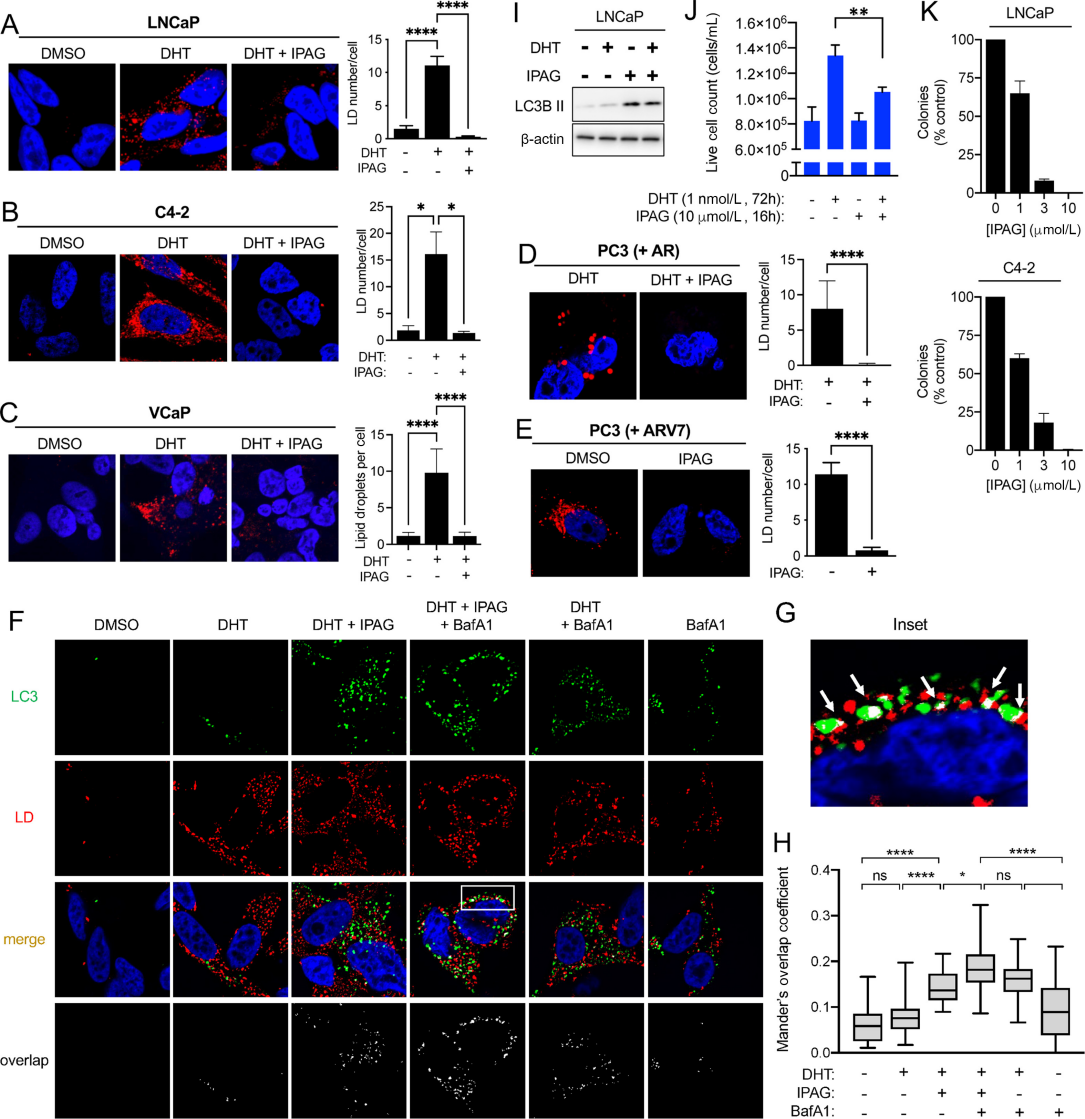
Pharmacologic Sigma1 inhibitor eliminates DHT-induced AR-mediated LDs by lipophagy. Treatment with a small-molecule Sigma1 inhibitor eliminates AR and ARV7 mediated LDs. Confocal image of HCS LipidTox stained LDs (red) in LNCaP cells (**A**), in C4-2 cells (**B**), and in VCaP cells (**C**) treated with drug vehicle (DMSO), DHT (1 nmol/L, 3 days), and treatment with DHT (1 nmol/L, 3 days) combined with Sigma1 inhibitor (IPAG, 10 µmol/L, added for the final 16 hours of the 3-day DHT treatment), DAPI stained nuclei (blue). Quantification of LDs expressed as the mean number of LDs per cell ± SEM. *, *P* < 0.05; **, *P* < 0.01; ***, *P* < 0.001; ****, *P* < 0.0001. **D,** Confocal image of LDs (red) in AR-transfected PC3 cells [PC3 (+AR)] treated with DHT (1 nmol/L, 3 days) and with Sigma1 inhibitor (IPAG, 10 µmol/L, added for the final 16 hours). **E,** ARV7-transfected PC3 cells [PC3 (+ARV7)] treated with drug vehicle (DMSO) and Sigma1 inhibitor (IPAG, 10 µmol/L, 16 hours). Quantification of LDs immediately to the right of micrographs. Data expressed as the mean number of particles per cell ± SEM. ****, *P* < 0.0001. **F,** Confocal micrographs showing colocalization of GFP-LC3 (LC3, green) and HCS LipidTox labeled LDs (red) in LNCaP (GFP-LC3) cells that were cultured in CSS medium for 3 days and treated with 1 nmol/L DHT for 3 days alone or in combination with 10 µmol/L IPAG and 10 nmol/L bafilomycin A1 (Baf A1) for the final 8 hours prior to fixing the cells. **G,** Inset from column 4, white boxed area, Merge from E showing magnified view of autophagosome (LC3, green) colocalization with LD (red). Overlapping, colocalization events indicated by white arrows. **H,** Box and whisker plot of Mander overlap coefficients. Data are presented as mean ± SEM from three independent experiments. *, *P* < 0.05; **, *P* < 0.01; ***, *P* < 0.001; ****, *P* < 0.0001; ns, no significance. **I,** Immunoblots of whole-cell protein extracts from parallel cell culture and experimental conditions used in J. **J,***In vitro* cell proliferation assay of LNCaP cells precultured in CSS medium for 3 days and then treated for 3 days with 1 nmol/L DHT and IPAG (10 µmol/L) was added for the final 16 hours. Live cells were counted by Trypan blue exclusion assay. Error bars represent SEM. **, *P* < 0.01. **K,** LNCaP and C4-2 colony formation is suppressed by IPAG in a dose-responsive manner. Data presented as relative number of colonies compared with no drug treatment (as % control).

We have published that a prototypic small-molecule Sigma1 inhibitor, IPAG, can be used to suppress aberrant AR signaling in prostate cancer cells (57), and we also showed that it could induce autophagy in several different cancer cell lines (55, 56). We therefore asked whether cotreatment with the Sigma1 inhibitor could suppress DHT induced LDs. We found that IPAG can eliminate DHT-induced LDs in LNCaP, C4-2, and VCaP cells (Fig. 5A–C). Thus, both RNAi mediated KD and pharmacologic inhibition of Sigma1 prevented androgen-induced LD accumulation, indicating that pharmacologic inhibition of Sigma1 can phenocopy features of Sigma1 KD (Figs. 2 and 5). Furthermore, we found that treatment with the small-molecule Sigma1 inhibitor could eliminate AR- and ARV7-induced LDs in transfected PC3 cells, an AR- and ARV-negative cell line, thereby confirming that the Sigma1 inhibitor eliminated both androgen-stimulated AR and constitutively active ARV7-induced LDs (Fig. 5D and E).

Of note, the Sigma1 inhibitor eliminated already formed LDs in all these conditions, suggesting that it triggered and/or accelerated LD degradation (Fig. 5A–E). We previously reported that IPAG, and other selective small-molecule Sigma1 inhibitors, could trigger autophagy and autophagic flux in several cancer cell lines (54–56). Therefore, we asked whether the elimination of LDs in response to Sigma1 inhibitor treatment was mediated by lipophagy. We found that IPAG treatment significantly increased autophagosome (LC3) numbers and correspondingly decreased the number of LDs in DHT-treated LNCaP cells (Fig. 5F). By 16 hours of IPAG treatment, nearly all LDs were eliminated, however, by 8 hours of IPAG treatment, LD elimination begins, and colocalization of autophagosomes and LDs was detectable (Fig. 5F and G). Cotreatment with IPAG and Baf A1 resulted in increased numbers of GFP-LC3–positive autophagosomes that colocalized with LDs (Fig. 5F and G). Baf A1-blocked Sigma1 inhibitor triggered degradation of DHT-induced LDs in LNCaP cells (Fig. 5F and G). Blocked autophagic flux corresponded with accumulation of autophagosomes (Fig. 5F and G). We quantified these LD-autophagosome colocalization events using Mander coefficient overlap correlation analysis (71), and we confirmed significant differences in overlap coefficient in treatment conditions where LD and autophagosome colocalization was observed, in particular in the IPAG treatment conditions (Fig. 5H). Altogether, these data demonstrate that pharmacologic inhibition of Sigma1 triggers lipophagy in prostate cancer cells and phenocopies key aspects of Sigma1 shRNA KD.

### Pharmacologic Inhibition of Sigma1 Suppresses Prostate Cancer Cell Proliferation Despite Lipophagy

We asked how Sigma1 inhibitor–induced lipophagy would impact prostate cancer cell proliferation. We found that IPAG blocked proliferation when added during the last 16 hours of a 72-hour DHT treatment time course (Fig. 5I and J). Under these conditions, DHT induced a modest increase in LC3B II levels, indicating modest increase in autophagy. IPAG treatment induced higher levels of LC3B II (Fig. 5I). In a longer-term measure of *in vitro* survival and proliferation, IPAG suppressed both LNCaP and C4-2 colony formation in a dose-responsive manner (Fig. 5K).

### Small-molecule Sigma1 Inhibitor Blocks Androgen-mediated Antioxidant Response and Redox Homeostasis

**FIGURE 6:**
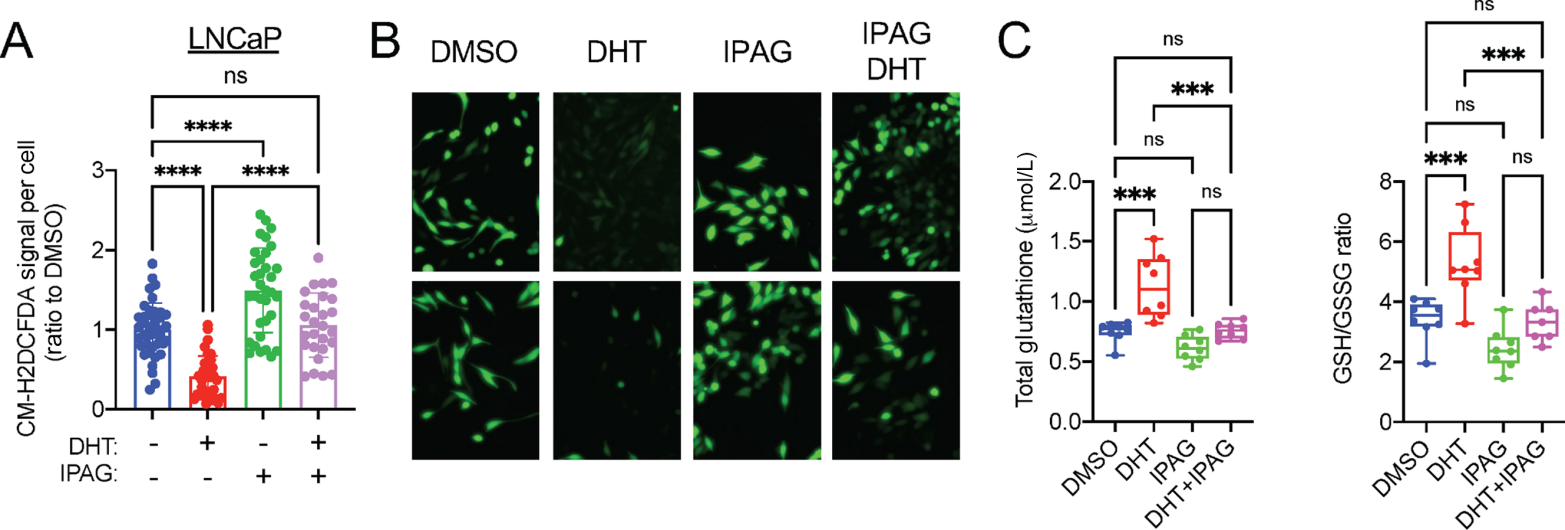
Sigma1 small-molecule inhibition disrupts GSH ratios and increases ROS levels in LNCaP and C4-2 cells. **A,** Quantification of CM-H_2_DCFDA signal per cell in LNCaP cells cultured in CSS medium for 3 days and treated for 3 days with DHT (1 nmol/L) alone or combined with 10 µmol/L IPAG for the last 16 hours. Data are presented as mean ± SEM from three independent experiments, *, *P* < 0.05; **, *P* < 0.01; ***, *P* < 0.001; ****, *P* < 0.0001; ns, no significance. **B,** Representative fluorescent micrographs showing CM-H_2_DCFDA levels in LNCaP cells in A. **C,** Total GSH and GSH:GSSG measurements in LNCaP cells treated as in A. Data are presented as mean ± SEM from three independent experiments. *, *P* < 0.05; **, *P* < 0.01; ***, *P* < 0.001; ****, *P* < 0.0001; ns, no significance.

Similar to Sigma1 KD, we found that treatment of LNCaP and VCaP cells with IPAG significantly increased ROS levels (CM-H2DCFDA signal per cell) and prevented DHT-mediated decrease in ROS (Fig. 6A and B for LNCaP and Supplementary Fig. S5 for VCaP cells). This corresponded with increased total GSH concentrations and reduced GSH/GSSG ratios in DHT-treated LNCaP cells, consistent with a redox homeostasis promoting effect of AR (Fig. 6C). The Sigma1 inhibitor suppressed DHT-induced total GSH and GSH/GSSG ratios, suggesting that pharmacologic inhibition of Sigma1 can disrupt androgen-AR axis–mediated antioxidant response (Fig. 6C).

In the cell proliferation experiments performed here, Sigma1 inhibition (by shRNA KD and treatment with a small-molecule inhibitor) suppressed DHT-induced proliferation, even in the presence of NAC (Supplementary Fig. S6). Although it is conceivable that an antioxidant such as NAC could quench and decrease excess ROS and restore productive levels of ROS induced by DHT, it is important to note that the exquisite control required to quench ROS to within the appropriate range required to promote DHT-induced proliferation is extremely difficult to implement simply with exogenous application of chemical quenching agents. Indeed, the biology of ROS is complex. There are multiple species of ROS that require tight control in concentration, time (as these molecules are extremely labile and reactive), and space (compartments within the cell; refs. 26–30, 33–38, 40).

### Prostate Tumors in Which *SIGMAR1* mRNA Transcripts are Elevated are Enriched in Gene Transcripts Involved in Lipid Metabolism and ROS-associated Pathways

**FIGURE 7:**
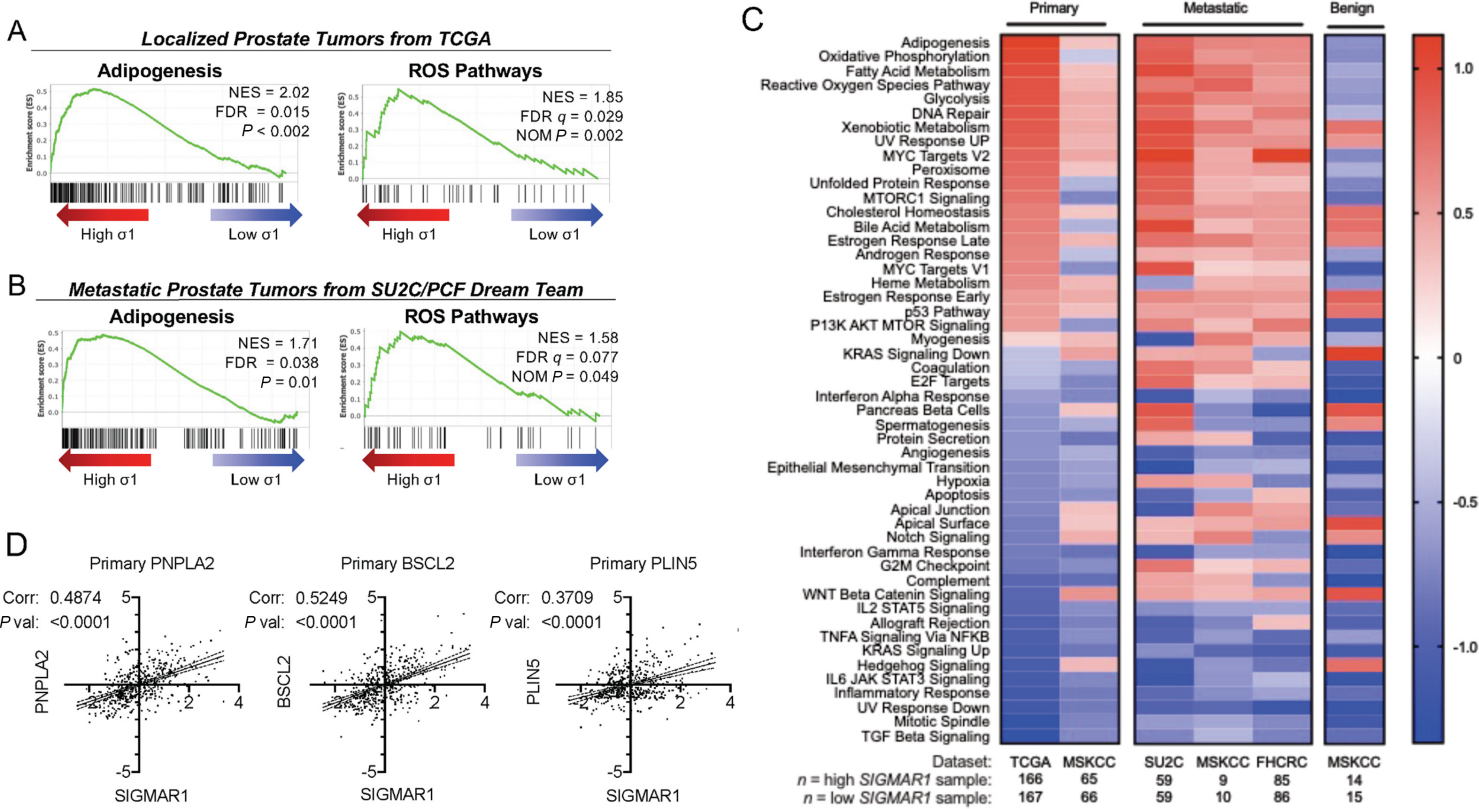
GSEA and correlation analysis of Sigma1/*SIGMAR1* in prostate tumors. GSEA using Adipogenesis and ROS Pathway Hallmark gene sets on localized prostate tumors from TCGA (**A**; 60) and metastatic prostate tumors from SU2C/PCF Dream Team (**B**; 61). **C,** Heat map of normalized enrichment scores from primary, metastatic, or benign prostate tissue utilizing TCGA, MSKCC, SU2C/PCF Dream Team, and FHCRC datasets (60, 61, 63). **D,** Single gene correlation analysis between *SIGMAR1* and *PNPLA2*, *BCSL2,* and *PLIN5* using Z-scores from published primary prostate tumor datasets (60, 63, 64).

GSEA revealed that *SIGMAR1* mRNA is enriched in tumor tissue with elevated adipogenesis and ROS pathway–associated genes in both localized and metastatic prostate tumors (Fig. 7A and B), however, not in adjacent benign prostate tissue (Fig. 7C). Among the gene transcripts that most saliently correlated with *SIGMAR1* mRNA were essential LD biogenesis and metabolism genes, *ATGL/PNPLA2*, *BSCL2*, and *PLIN5* (Fig. 7D). Of note, *ATGL/PNPLA2* plays a central regulatory role in LD lipolysis, and it also has been reported to mediate/activate lipophagy in the liver, in part by promoting interactions between LC3 and LDs and subsequent autophagic flux (4, 73). Consistent with this notion, ATGL and PLIN5 proteins levels increased with prolonged DHT treatment of LNCaP cells (Supplementary Fig. S7).

## Discussion

### AR-driven Metabolism and Adaptive Resistance Mechanisms

Aberrant AR signaling drives multiple aspects of prostate cancer cell metabolism, growth, proliferation, and cellular plasticity (44). Whereas in normal prostate cells, androgens drive anabolic lipid metabolism to support the secretory function of the prostate gland, the dysregulated lipid metabolism associated with aberrant AR signaling in prostate cancer contributes to proliferation, disease progression, and the development treatment resistance (reviewed in ref. 44). Emerging lines of evidence suggest a crucial role of downstream and convergent cellular pathways such as autophagy in AR-driven prostate cancer biology. Genes involved in lysosomal biogenesis and function as well as core autophagy genes were recently identified as transcriptional targets of AR in prostate cancer (74). Moreover, androgen-stimulated/AR-mediated autophagy was shown to promote cell growth and proliferation of prostate cancer cells by augmenting intracellular lipid accumulation into LDs (13).

**FIGURE 8:**
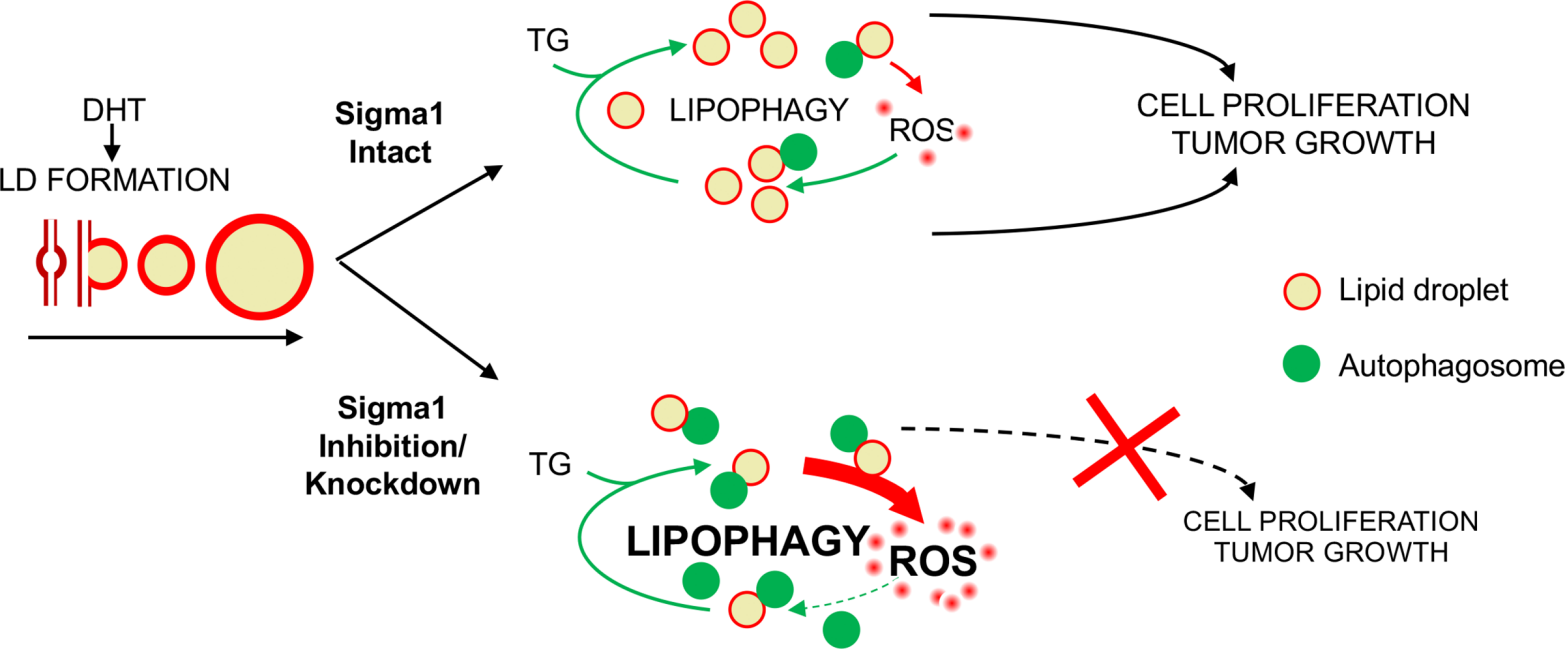
Working Model. Sigma1 targeting disrupts LD-mediated redox homeostasis in prostate cancer cells. LD, lipid droplet; TG, triacylglycerol; DHT, dihydrotestosterone; ROS, reactive oxygen species.

However, the range of mechanisms by which AR controls physiologic signaling networks, how they are dysregulated during disease progression, and the complex network of pathways underlying the emergence of resistant prostate cancer are not fully understood (44, 75, 76). Metabolic plasticity reflects the cooperative convergence of multiple pathways including autophagy, lipid metabolism, and cellular redox homeostasis and oxidative stress response mechanisms. Our data suggest that Sigma1 is a regulator at the intersection of these pathways in prostate cancer cells. We previously reported a physical and functional association between AR and Sigma1 (57). Here, we demonstrate that androgen-induced, AR-mediated LD metabolism and the autophagy that promote prostate cancer cell proliferation requires Sigma1 and is downstream of AR signaling. This work suggests a novel physiologic role for Sigma1 in regulating lipid metabolism and redox homeostasis pathways to promote the metabolic plasticity that enables prostate cancer cell proliferation (Fig. 8).

LDs are emerging as important contributors to tumor metabolism and oxidative stress response. LDs promote tumorigenesis through storage, transport, and distribution of fatty acids and lipids (77). By buffering against oxidative stress, LD accumulation also promotes the cellular redox homeostasis crucial for cancer cell survival, growth, and proliferation (77). LD accumulation and stability are determined by lipolysis or lipophagy (2, 78, 79). Endosomal and autophagosomal vesicle trafficking pathways converge on lysosomes, which coordinate sorting and distribution of both exogenous and endogenous lipids to various organelles and cellular membrane compartments (80). LDs can be trafficked to lysosomes by autophagosomes and degraded by lipophagy (2, 78, 79).

Cross-talk between LDs, lysosomes, and the endoplasmic reticulum (ER) serves to regulate cellular distribution of sterols and fatty acids and metabolic homeostasis, and LD–lysosome interactions are involved in ER stress responses (3, 81). In prostate cancer, LDs have been associated with aggressive disease and are thought to support the aberrant lipid metabolism and adaptive resistance that contributes to disease progression (5, 79, 82). Experimental cancer models to investigate the role of LDs in tumor biology and cancer progression support these clinical observations. For example, a recent publication reported elevated levels of intratumoral LDs in mice fed high-fat diet associated with aggressive tumor growth and metastasis (82). The biogenesis, activity, and degradation of LDs is a highly orchestrated process; however, the factors involved in this process, particularly in the context of prostate cancer, remain poorly defined (1, 2).

Sigma1 is a novel ligand-operated scaffolding or chaperone protein that supports the increased demand for lipid and protein synthesis associated with tumor growth (54). In this study, we have discovered a novel and specific role for Sigma1 in LD metabolism. Sigma1 has been shown to physically associate with and contribute to remodeling of lipid microdomains in the ER membrane (83, 84). In a study using NG108 cells, Sigma1 was reported to colocalize with nascent LDs on the endoplasmic reticulum (ER-LD) membrane, prior to budding into the cytosol (83), implicating Sigma1 in the compartmentalization and distribution of membrane-associated lipids. We previously demonstrated that small-molecule modulators of Sigma1 can induce selective autophagy via an ER stress–associated mechanism (55, 56). Here, we extend our findings and show that pharmacologic modulation of Sigma1 in prostate cancer cells can trigger the degradation of LDs by an autolysosomal degradation mechanism consistent with lipophagy, likely via ER stress–associated autophagosome formation and subsequent autolysosomal degradation of LDs. The enhanced degradation of LDs following Sigma1 inhibition means LDs are no longer available to shuttle oncogenic lipids and proteins, nor are they available to buffer ROS levels. The loss of lipid stores and hubs for lipid metabolic processes along with oxidative stress associated with depletion of LDs suggests both decreased LD biogenesis and increased LD degradation contribute to the antitumor mechanism of Sigma1 inhibition.

Sigma1 as a novel regulator of lipid metabolism and redox homeostasis in prostate cancer. The effects of autophagy on cellular processes are context dependent and the double-edged nature of autophagy is common to many biological processes. In cancer cells, selective autophagy in controlled and limited amounts can fuel multiple metabolic pathways including glycolysis, glutaminolysis, and mitochondrial oxidative phosphorylation and beta-oxidation (8, 85). However, this benefit can be disrupted by excessive autophagy.

Maintaining redox homeostasis is also crucial to tumor growth and survival, and it is similarly highly dependent on dose and context. Whereas excessive ROS triggers proliferation arrest and cell death, in limited and localized quantities, ROS mediates cellular signaling that promotes survival and proliferation of cancer cells (29, 30, 40). With the goal of disrupting redox balance, rather than a simple positive or negative effect of ROS, emerging redox targeting cancer therapies now focus on acutely elevating ROS and overwhelming cancer cells and pushing cancer cells “over the edge” (40). We demonstrate a role for Sigma1 in this context.

Sigma1 serves a support role in tumor biology. Sigma1 does not drive, but rather enables tumor-promoting processes. We propose that Sigma1 serves as a regulatory hub at the intersection of an AR-driven autophagy-LD-oxidative stress response in cancer cells. Sigma1 inhibitors may serve a dual purpose of inducing oxidative stress by limiting LD-mediated ROS buffering while also inhibiting ER-mediated stress response pathways. This may restrict the metabolic plasticity and adaptive capacity of cancer cells and thus prevent the rewiring that enables resistance to therapies that impact tumor metabolism (86).

## Supplementary Material

Figure S1 Mander Coefficient of LD-auophagosome overlap in Sigma1 shR KD cells.

Figure S2 ATG5 and 7 shRNA KD

Figure S3 VCaP data

Figure S4 VCaP and C4-2 ROS LD data

Figure S5 VCaP ROS and autophagy

Figure S6 LNCaP DHT + NAC + Sigma1 shR proliferation

Figure S7 ATGL PLIN5 induction
