# Supplementary material for: Sigma1 Regulates Lipid Droplet–Mediated Redox Homeostasis Required for Prostate Cancer Proliferation
Source: Cancer Res Commun. 2023 Oct 30;3(10):2195–210. doi: 10.1158/2767-9764.CRC-22-0371 (PMC10615122; doi:10.1158/2767-9764.CRC-22-0371)
Supplement: Figure S1 — Mander Coefficient of LD-auophagosome overlap in Sigma1 shR KD cells. [file crc-22-0371-s01.pdf]

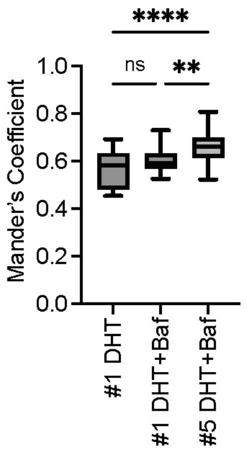

Supplemental Figure 1. Mander's Overlap Coefficient of LD and autophagosome overlap in DHT and bafilomycin A1 (BafA1) treated LNCaP cells. Treatment conditions and shRNA are described in Figure 2. Data are presented as mean ± SEM. **p* < 0.05, ** *p* < 0.01, *****p* < 0.0001, ns = no significance.
