# Supplementary material for: Sigma1 Regulates Lipid Droplet–Mediated Redox Homeostasis Required for Prostate Cancer Proliferation
Source: Cancer Res Commun. 2023 Oct 30;3(10):2195–210. doi: 10.1158/2767-9764.CRC-22-0371 (PMC10615122; doi:10.1158/2767-9764.CRC-22-0371)
Supplement: Figure S2 — ATG5 and 7 shRNA KD [file crc-22-0371-s02.pdf]

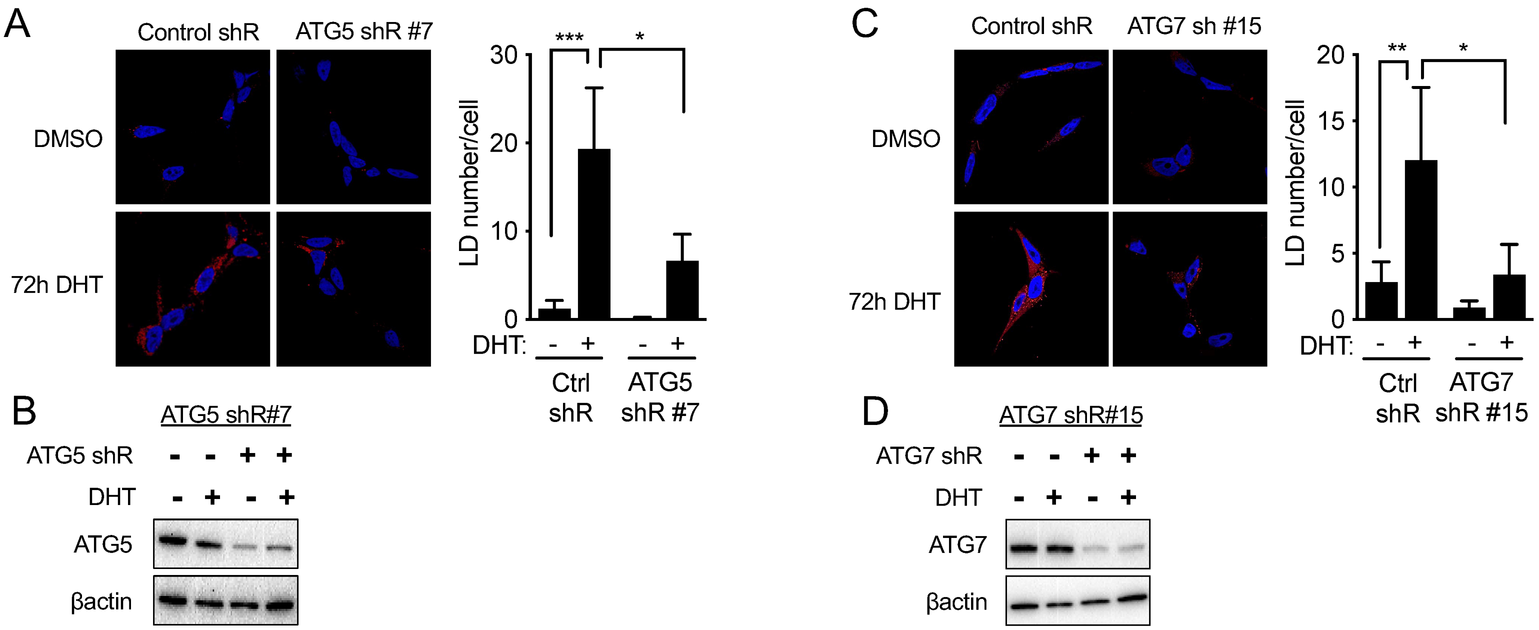

Supplemental Figure 2. Essential autophagy genes, ATG5 and ATG7, are required for androgen induced LD accumulation. (A) LDs (red) in LNCaP cells transduced with non-specific control shRNA or ATG5 shRNA, clone #7 and subsequently treated with drug vehicle (DMSO, 72 hours), or DHT (1 nM, 72 hours). DAPI stained nuclei (blue). Quantification of LD numbers per cell under these conditions, panel to right. Data expressed as the mean number of particles per cell ± SE. **p* < 0.05, ****p* < 0.001**. (B)** Immunoblot confirmation of ATG5 shRNA knockdown in (A). **(C)** LDs (red) in LNCaP cells transduced with non-specific control shRNA or ATG7 shRNA, clone #15 and subsequently treated with drug vehicle (DMSO, 72 hours) or DHT (1 nM, 72 hours). DAPI stained nuclei (blue). Quantification of LD numbers per cell under these conditions, panel to right. Data expressed as the mean number of particles per cell ± SE. **p* < 0.05, ****p* < 0.001, *****p* < 0.0001**. (D)** Immunoblot confirmation of ATG7 shRNA knockdown in (C).
