# Supplementary material for: Sigma1 Regulates Lipid Droplet–Mediated Redox Homeostasis Required for Prostate Cancer Proliferation
Source: Cancer Res Commun. 2023 Oct 30;3(10):2195–210. doi: 10.1158/2767-9764.CRC-22-0371 (PMC10615122; doi:10.1158/2767-9764.CRC-22-0371)
Supplement: Figure S3 — VCaP data [file crc-22-0371-s03.pdf]

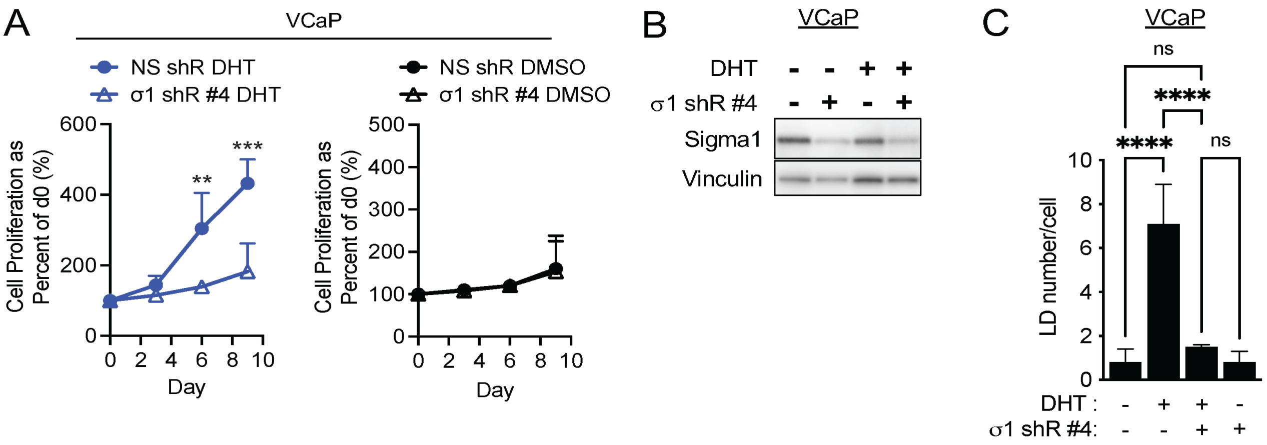

Supplemental Figure 3. Sigma1 knockdown suppresses DHT induced VCaP cell proliferation and tumor growth despite (or due to) increased autophagy and decreased LDs. (A) In vitro cell proliferation assay of Sigma1 shRNA transduced VCaP cells pre-cultured in CSS medium for 3 days and then treated for 3, 6, and 9 days with 1 nM DHT. Data are represented as fold induction over cells treated with control shRNA at day 0 (Percent of d0). ** $p < 0.01$. *** $p < 0.001$. **(B)** Immunoblots of whole-cell protein extracts from parallel VCaP cell culture performed in parallel and using same experimental treatment conditions as in (A). Data shown for Sigma1 shRNA clone #4 knockdown VCaP cell cultures. **(C)** VCaP cells treated as described above in (A). Quantification of LD number per cell and average area of LD number/cell, as described above. Data represent mean values from at least 3 independent determinations, and error bars represent SEM. Statistical analysis was performed using ANOVA and Bonferroni post-test. ****$p < 0.0001$, ns = no significance.
