# Supplementary material for: Sigma1 Regulates Lipid Droplet–Mediated Redox Homeostasis Required for Prostate Cancer Proliferation
Source: Cancer Res Commun. 2023 Oct 30;3(10):2195–210. doi: 10.1158/2767-9764.CRC-22-0371 (PMC10615122; doi:10.1158/2767-9764.CRC-22-0371)
Supplement: Figure S4 — VCaP and C4-2 ROS LD data [file crc-22-0371-s04.pdf]

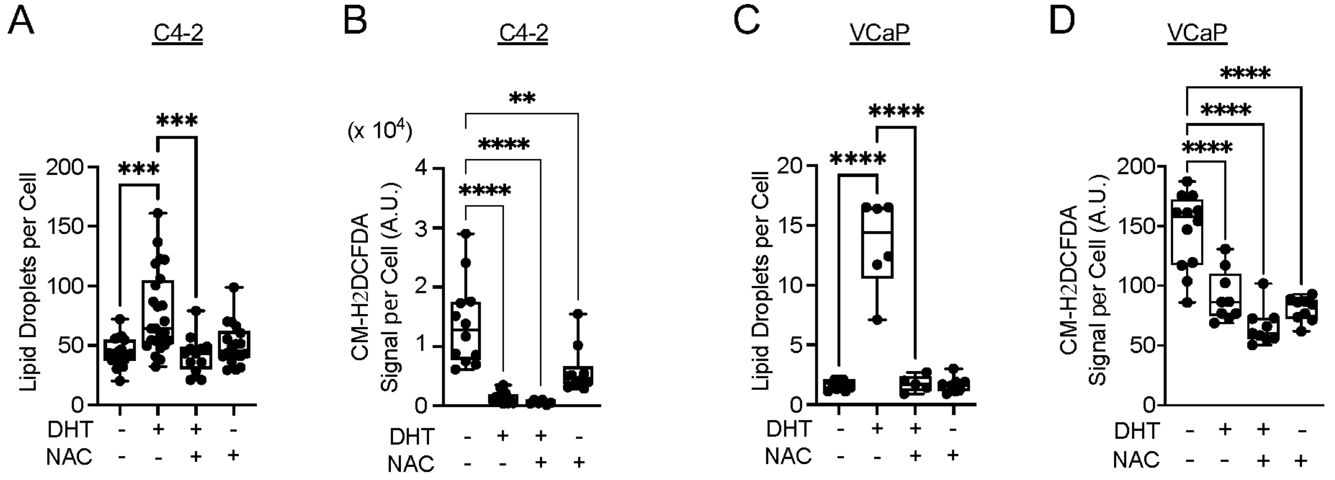

Supplemental Figure 4. LDs as buffers of DHT induced ROS and DHT promotes ROS homeostasis in C4-2 and VCaP cells. (A) LD quantification in C4-2 cells cultured in CSS containing medium for 3 days and treated with DMSO (vehicle) and 1 nM DHT alone or combined with 2.5 mM N-acetyl-L-cysteine (NAC) for 3 days. Data represent LDs per cell. **(B)** Quantification of reactive oxygen species (ROS), detected with CM-H_2DCFDA in C4-2 treated as described above in (A). Data are presented as mean ± SEM from at least 3 independent determinations. *$p < 0.05$, ** $p < 0.01$, ***$p < 0.001$, ****$p < 0.0001$. **(C)** LD quantification in VCaP cells cultured in CSS containing medium for 3 days and treated with DMSO (vehicle) and 1 nM DHT alone or combined with 2.5 mM N-acetyl-L-cysteine (NAC) for 3 days. Data represent LDs per cell. **(D)** Quantification of reactive oxygen species (ROS), detected with CM-H_2DCFDA in VCaP treated as described above in (A). Data are presented as mean ± SEM from at least 3 independent determinations. *$p <$ 0.05, ** $p < 0.01$, ***$p < 0.001$, ****$p < 0.0001$.
