# Supplementary figures and images for: Sigma1 Regulates Lipid Droplet–Mediated Redox Homeostasis Required for Prostate Cancer Proliferation
Source: Cancer Res Commun. 2023 Oct 30;3(10):2195–210. doi: 10.1158/2767-9764.CRC-22-0371 (PMC10615122; doi:10.1158/2767-9764.CRC-22-0371)

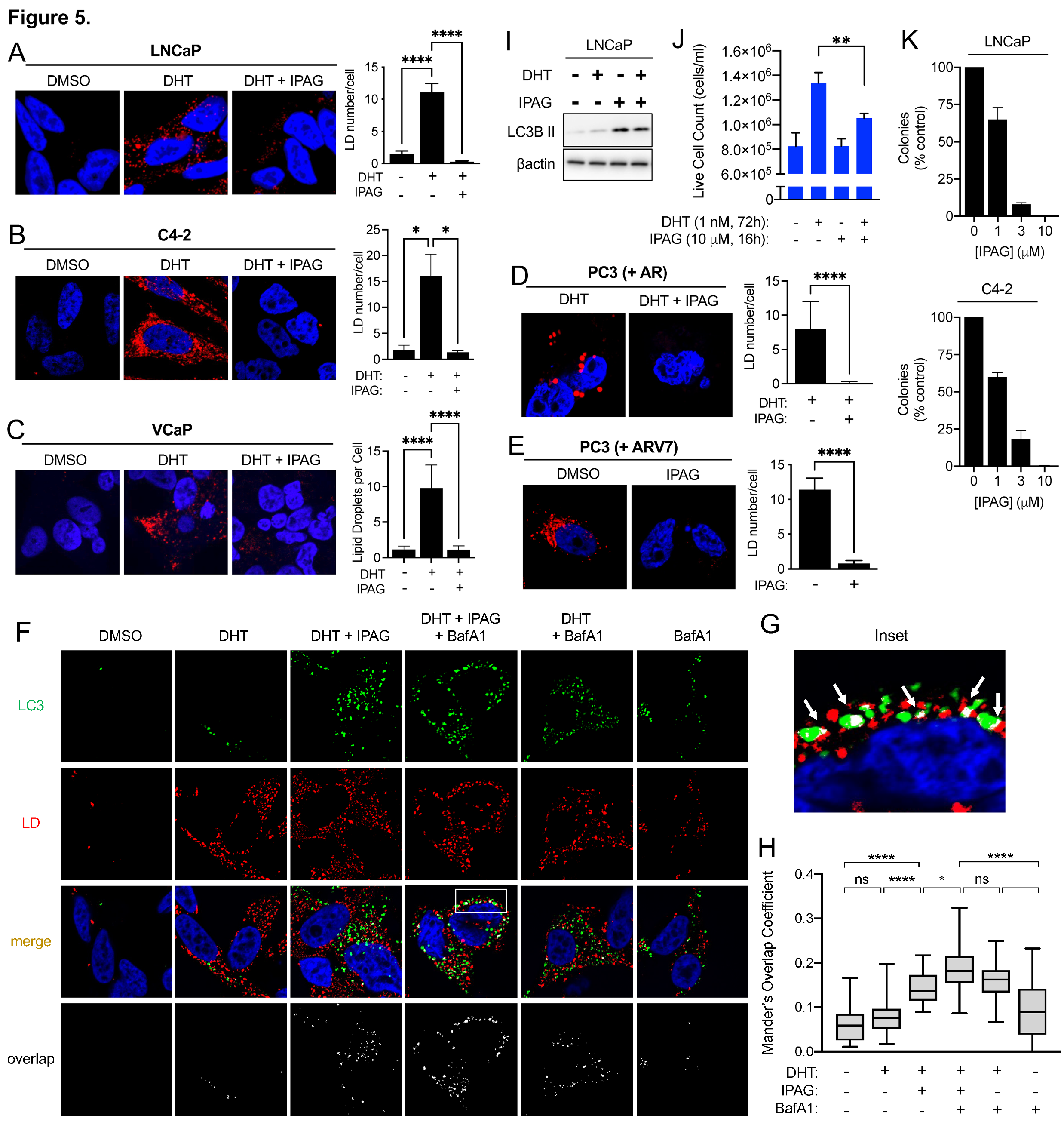

Supplement: Figure S5 — VCaP ROS and autophagy [file crc-22-0371-s05.png]

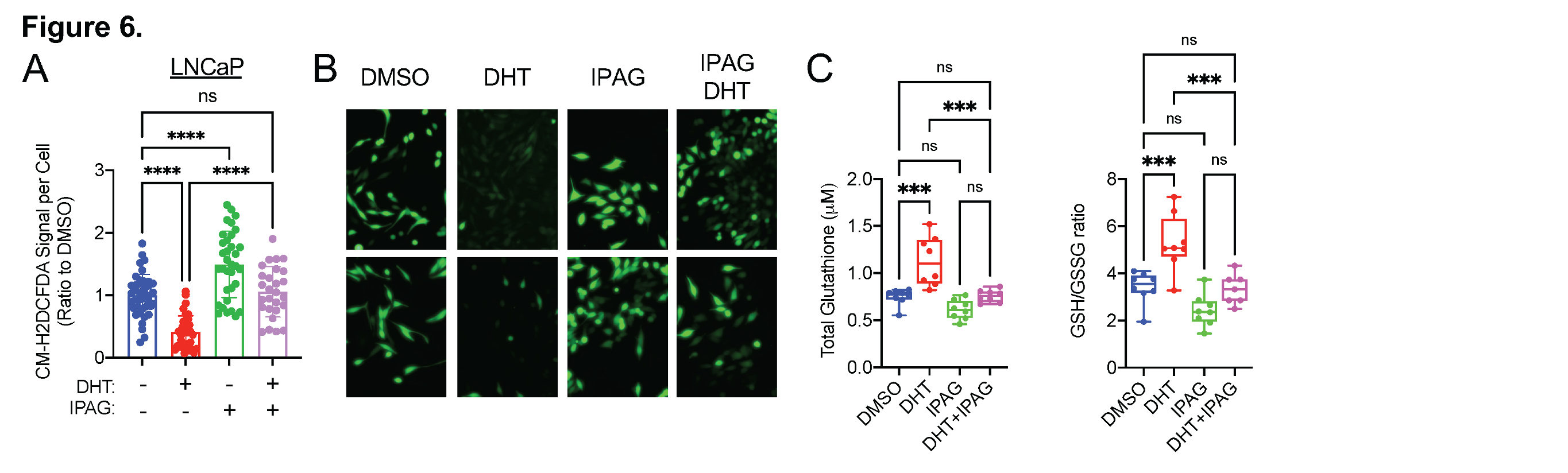

Supplement: Figure S6 — LNCaP DHT + NAC + Sigma1 shR proliferation [file crc-22-0371-s06.png]

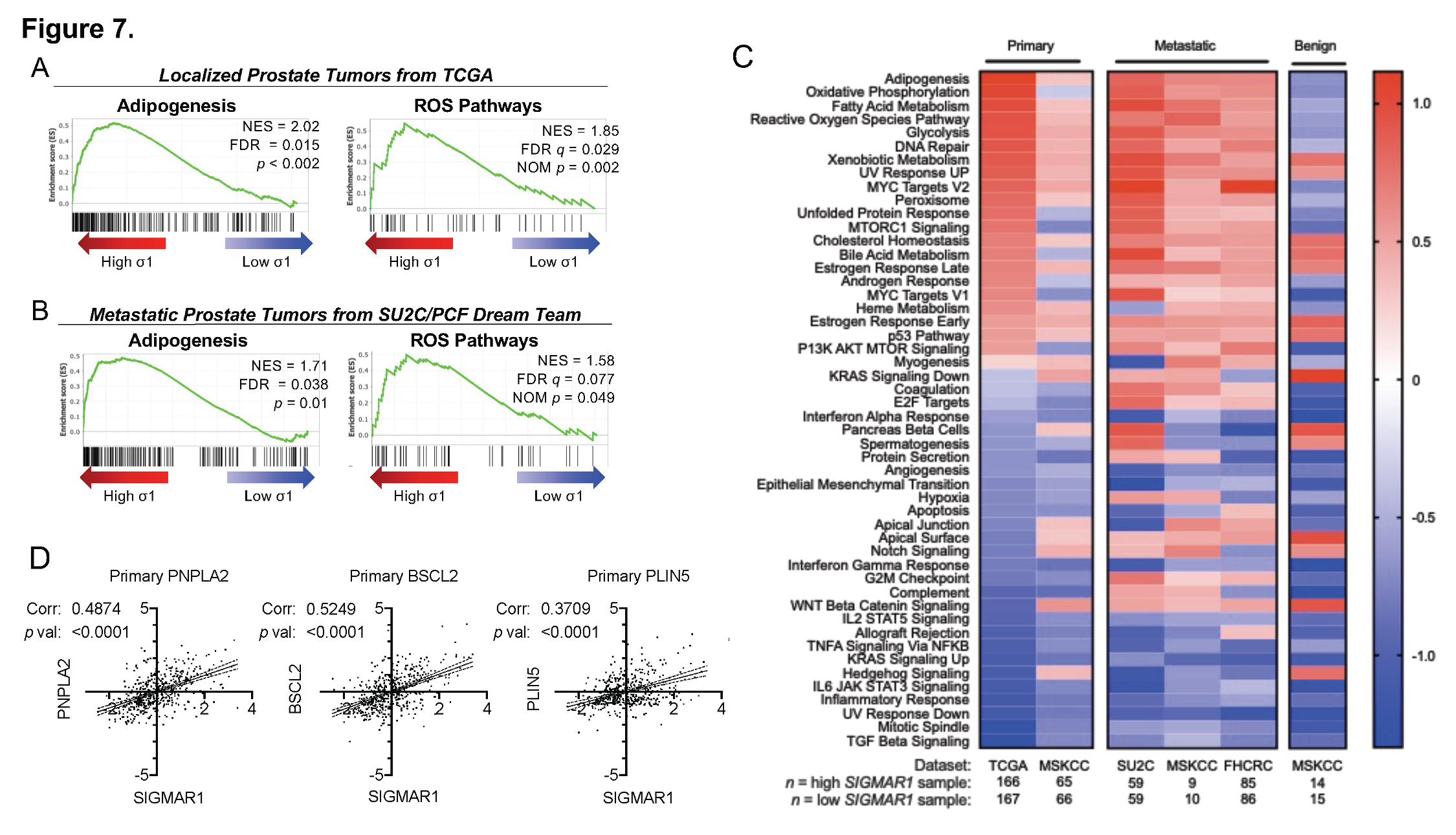

Supplement: Figure S7 — ATGL PLIN5 induction [file crc-22-0371-s07.png]
